# Potential biomarkers for early periodontal inflammation: investigating CD5^+^ B cells, salivary cytokines and oral microbiome

**DOI:** 10.1038/s41598-026-37044-6

**Published:** 2026-02-18

**Authors:** Elisabeth Clara Gottschalk, Oleksandra Chabanovska, Praveen Vasudevan, Israel Barrantes, Bernd Kreikemeyer, Wendy Bergmann-Ewert, Robby Engelmann, Brigitte Müller-Hilke, Hermann Lang

**Affiliations:** 1 Department of Operative Dentistry and Periodontology, University Medical Center Rostock, Rostock, Germany; 2 Research Group Translational Bioinformatics, Institute for Biostatistics and Informatics in Medicine and Ageing Research, University Medical Center Rostock, Rostock, Germany; 3 Institute of Medical Microbiology, Virology and Hygiene, University Medical Center Rostock, Rostock, Germany; 4 Core Facility for Cell Sorting and Cell Analysis, University Medical Center Rostock, Rostock, Germany; 5 Clinic III (Hematology, Oncology and Palliative Medicine), Special Hematology Laboratory, Rostock University Medical School, Rostock, Germany

**Keywords:** CD5^+^ B cells, Cytokines, Oral microbiome, Periodontal health, Periodontitis, Periodontitis, Biomarkers, Microbiome

## Abstract

Periodontitis is a highly prevalent chronic inflammatory disease that causes progressive destruction of the tooth-supporting apparatus and is linked to multiple systemic disorders. Despite its high prevalence, early biomarkers capable of predicting individual susceptibility remain elusive. The multifactorial nature of the disease and the lack of a validated, specific indicators hinder reliable early diagnosis before irreversible tissue damage occurs. CD5^+^ B cells, known for their autoreactive potential and role in bone resorption, have been previously found elevated in advanced periodontitis. Due to their association with tissue destruction, these cells may hold predictive value even at earlier stages. However, their systemic relevance in early periodontal inflammation is scarcely explored. This cross-sectional study examined differential subsets of circulating CD5^+^ B cells as potential systemic biomarkers of early periodontal disease. Sixty patients were grouped as healthy, gingivitis and moderate chronic periodontitis. Multiple regression analysis revealed that besides age (*p* = 0.009), the double negative (CD27^−^IgD^−^) subset of CD5^+^ memory B cells was related to periodontitis, while total CD5^+^ B cell levels remained unchanged. To characterize the oral inflammatory milieu, salivary cytokines and the composition of the subgingival microbiome were analyzed. Salivary IL-8 levels and IL-17A detection rates were significantly elevated in periodontitis. Microbiome profiling further identified an exploratory correlation between *Megasphaera* and salivary IL-8 in gingivitis, which may represent an early-stage signal but requires validation in larger follow-up cohorts.

**Supplementary Information:**

The online version contains supplementary material available at 10.1038/s41598-026-37044-6.

## Introduction

Periodontitis is a widespread dental disease with an estimated global prevalence rate of 10%, which is characterized by a progressive destruction of the tooth-supporting apparatus due to a chronic inflammation in periodontium^1^. According to the 2014 Oral Health Study^2^, every fifth young adult (35–44 years) and every second younger senior (65–74 years) in Germany has moderate chronic periodontitis. Chronic periodontitis is also associated with multiple systemic dysfunctions including cardiovascular, metabolic, inflammatory and autoimmune diseases, cancer, systemic bone loss as well as obstetric complications^3^. The biological origin behind these reported associations appears to lie in the disrupted interplay between periodontal microbiota and the host immune system, leading to immunological dysregulation^4^. During the transition from a healthy to an advanced pathological state, the abundance of microbial by-products and cellular components in the gingival lesion gradually increases^5^. Once these antigens are recognized by host cells, the production of pro-inflammatory mediators is initiated^6,7^. In particular, the release of interleukin-1 beta (IL-1β), interleukin-6 (IL-6) and interleukin-8 (IL-8, CXCL8) plays a significant role in immunological response. The signaling cascade triggered by these cytokines ultimately leads to irreversible bone loss, the hallmark of periodontitis^8,9^.

Unfortunately, factors that would accurately predict patient’s susceptibility to periodontitis are elusive. While some known risk factors such as smoking or diabetes mellitus might account for periodontitis development^10–12^, the critical determinant for disease progression is probably the extent of immune response to the dysbiosis. A closer look to the local inflammation site revealed a significant expansion of B cells and plasma cells in advanced periodontitis^13^.

In this context, the role of CD5^+^ B cells, a subtype of CD19^+^ B cells, in severe periodontitis has been put into perspective over the last twenty years. In 2002, Berglundh et al*.*^14^ demonstrated considerably elevated population of CD5^+^ B cells in patients with aggressive local periodontitis. In 2009, the same research group indicated that nearly 60% of analyzed overall B cells (including CD5^+^ fraction) in chronic periodontitis lesion exhibited autoreactive properties^15^. In fact, the CD5^+^ B cells were linked to production of anti-collagen antibodies in inflamed gum as reported by Sugawara et al*.* in 1992^16^. The role of CD5^+^ B cells was also investigated in another instance of pathological bone resorption seen in patients suffering from rheumatoid arthritis (RA), where dysregulated immune response similarly leads to tissue destruction. In RA patients, Engelmann et al*.*^17^ evidenced a positive correlation between frequency of CD5^+^ B cells in peripheral blood and serum levels of carboxy-terminal telopeptide of type 1 collagen, which is a common biomarker of bone resorption. Interestingly, a comprehensive review provided by González-Febles and Sanz^18^ elucidates connection between periodontitis and RA in humans, thereby the first may affect the RA development but not vice versa.

Considering the described autoreactivity of CD5^+^ B cells as well as their potential to facilitate bone resorption, the extent of CD5^+^ cell expansion could possibly forecast the course of periodontal disease. However, in context of periodontal health, the CD5^+^ B cell involvement was examined predominantly in severe cases of periodontitis lacking broad microbiological background. In this study, we aimed to investigate CD5^+^ B lymphocytes involvement in chronic moderate periodontitis compared to gingivitis and healthy subjects with respect to CD5^+^ cell maturation, salivary cytokine expression as well as subgingival microbiota composition.

## Results

### Clinical characteristics of study groups

**Fig. 1:**
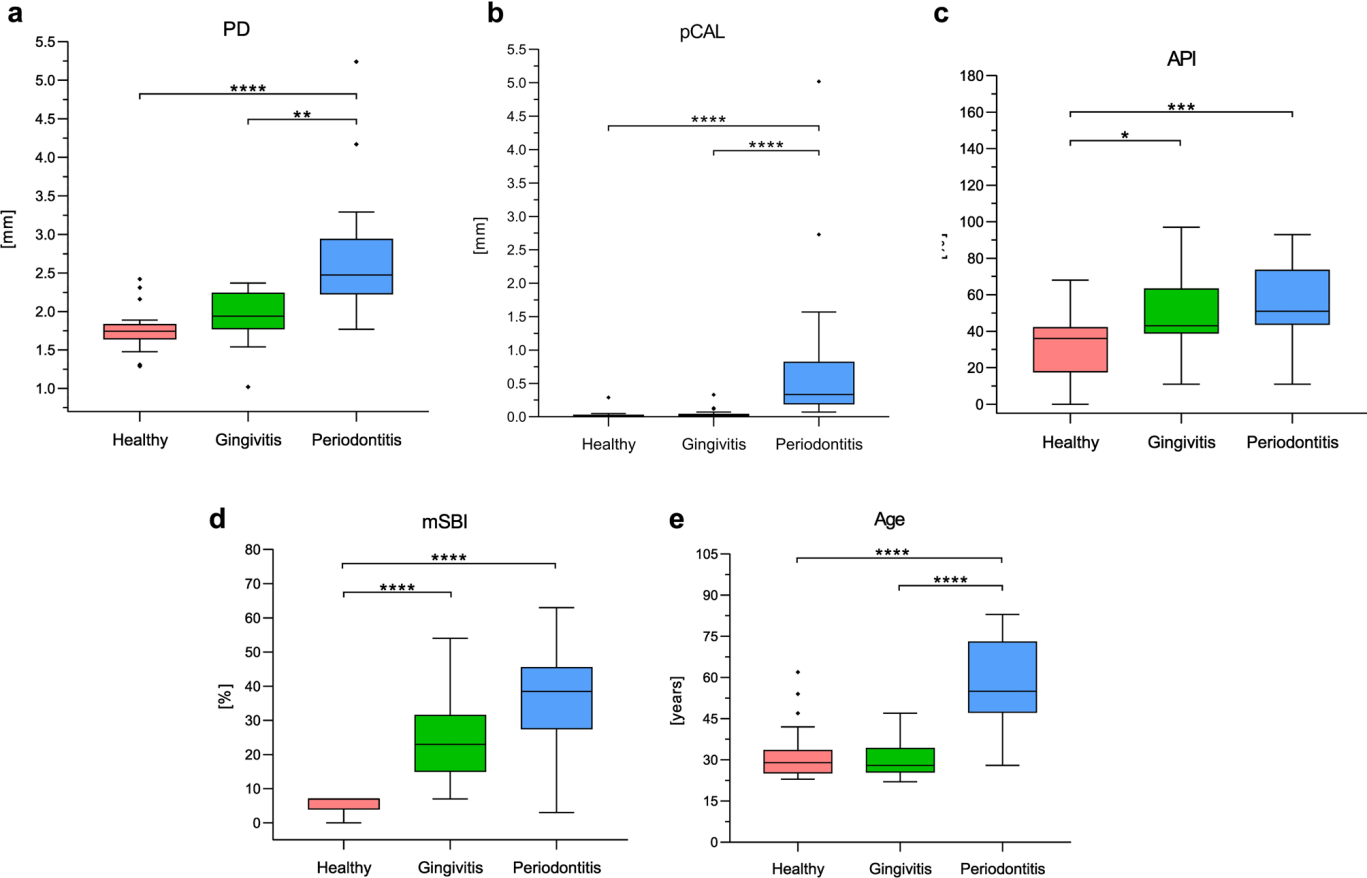
Classification of patients based on clinical assessment of periodontal health. (**a**) Probing depth (PD), (**b**) proxy clinical attachment level (pCAL), (**c**) approximal plaque index (API) and (**d**) modified sulcus bleeding index (mSBI) were assessed in patients to classify their dental status. (**e**) Additionally, age distribution across the groups is visualized. The box-and-whisker plots indicate the median value (horizontal line), the interquartile range (IQR as bottom and top of the box) and the whiskers extending to the extreme values within 1.5xIQR. Points beyond the whiskers are outliers as defined by the Tukey method. N = 20 patients in each group. Significance was determined using Kruskal–Wallis test by Dunn’s correction for multiple comparisons (* *p* < 0.05, ** *p* < 0.01, *** *p* < 0.001 and **** p < 0.0001).

Study groups were categorized based on evaluation of probing depth (PD) and modified sulcus bleeding index (mSBI). Additional clinical parameters, including approximal plaque index (API) and a proxy clinical attachment level (pCAL) were recorded for descriptive characterization of the cohort but did not contribute to group classification. Descriptive statistics are summarized in Supplementary Tables S1, S2. The lowest mean PD (mm) was observed in healthy group (1.75 ± 0.3), followed by gingivitis (1.94 ± 0.33) and periodontitis with significant pocket deepening (2.71 ± 0.80; *p* < 0.0001 and *p* < 0.01 vs. healthy and gingivitis, respectively; Fig. 1a). A similar trend was observed for pCAL (mm) with periodontitis showing a significant increase in mean attachment loss (0.79 ± 1.19) compared to both health (0.03 ± 0.06) and gingivitis (0.05 ± 0.09; *p* < 0.001; Fig. 1b). The proportion of sites with PD ≥ 4 mm increased from 0.9 ± 1.0% in healthy and 1.1 ± 1.3% in gingivitis to 19.1 ± 17.4% in periodontitis, which also showed 4.2 ± 9.9% of sites with PD ≥ 6 mm. Consistent with this pattern, sites with pCAL ≥ 3 mm were almost absent in healthy (0.06 ± 0.29%) and gingivitis (0.25 ± 1.05%) but reached 10.97 ± 22.08% in periodontitis. Only periodontitis group showed sites with pCAL ≥ 5 mm (2.72 ± 8.30%). API (%) levels were significantly raised in diseased groups (healthy vs. gingivitis vs. periodontitis: 33.35 ± 18.07 vs. 49.60 ± 19.74 vs. 56.40 ± 22.13). The difference was statistically significant between the healthy and gingivitis groups (*p* < 0.05) and even more pronounced between the healthy and periodontitis groups (*p* < 0.001; Fig. 1c). The mSBI values were fivefold higher in gingivitis and sevenfold higher in periodontitis group (*p* < 0.001; Fig. 1d).

The healthy cohort had a mean age of 32.5 ± 10.6 years (11 females and 9 males; Fig. 1e). The gingivitis group had a comparable age of 30.9 ± 7.9 years (11 females and 9 males). Periodontitis patients were significantly older (58.5 ± 15.5 years; *p* < 0.0001) and the group consisted of 8 females and 12 males. Regression analysis confirmed that increasing age was a significant risk factor for periodontitis (OR = 1.30 with 95% CI [1.07–1.58]; *p* = 0.009), but no significant association was found with gender (*p* = 0.134).

### Impact of periodontal health on cytokine profiles

**Fig. 2:**
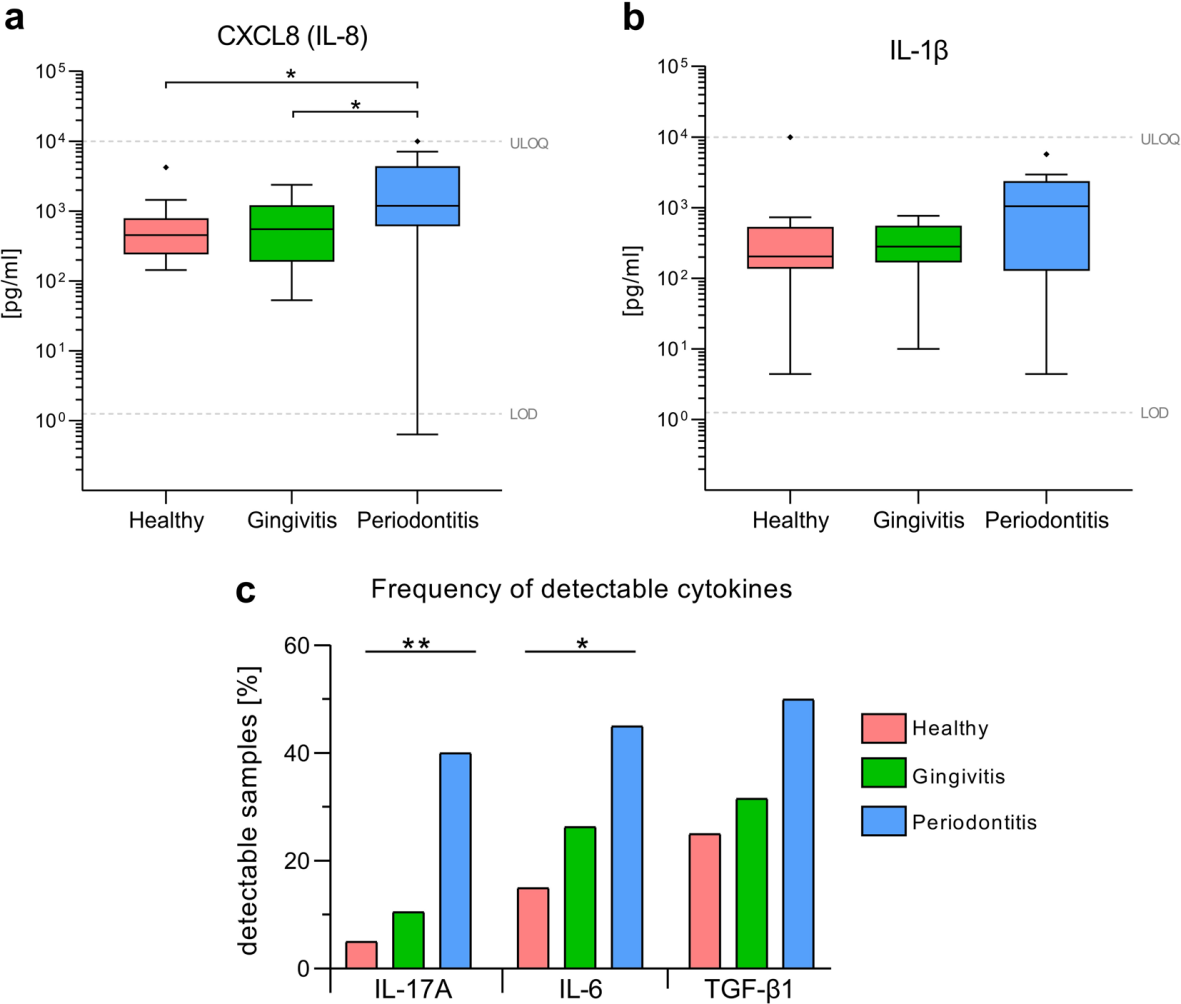
Salivary cytokine levels and detection frequencies in healthy and diseased groups. Presented are concentrations of (**a**) CXCL8 (IL-8) and (**b**) IL-1β in each group. The box-and-whisker plots indicate the median value (horizontal line), the interquartile range (IQR as bottom and top of the box) and the whiskers extending to the extreme values within 1.5xIQR. Points beyond the whiskers are outliers as defined by the Tukey method. Dashed horizontal lines indicate the assay’s lower limit of detection (LOD) and upper limit of quantification (ULOQ). Values below the LOD were imputed as LOD/2. Significance was determined using Kruskal–Wallis test followed by Dunn’s correction for multiple comparisons (* *p* < 0.05). (**c**) The bar chart depicts the percentage of samples with detectable IL-17A, IL-6 and TGF-β1 in each group. Group differences assessed by Chi-square test for trend are presented (*p < 0.05, **p < 0.01). Data in all panels (**a**–**c**) are from the same cohorts: healthy (n = 20), gingivitis (n = 19) and periodontitis (n = 20).

To assess the degree of inflammation in diseased groups, the salivary cytokine levels were examined. Measurable concentrations of IL-1β, CXCL8 (IL-8), CCL2 and CXCL10 could be detected in > 95% of analyzed samples (Supplementary Table S4). The comparison between groups revealed a significant twofold elevation of median CXL8 (IL-8) concentration in saliva of periodontitis patients compared to the healthy (*p* = 0.021) and gingivitis subjects (*p* = 0.047; Fig. 2a). Age-adjusted analysis confirmed these differences (Supplementary Table S5). For IL-1β, the overall difference between groups was borderline significant (global unadjusted *p* = 0.052; age-adjusted *p* = 0.051), with a trend toward higher medians in periodontitis (5.1-fold vs. healthy, *p* = 0.067; 3.7-fold vs. gingivitis, *p* = 0.195; Fig. 2b). No significant differences were observed for CCL2 or CXCL10.

Cytokines with less than 60% of samples showing detectable concentrations were analyzed as binary outcomes (detectable vs. non-detectable; Supplementary Table S6). Detection of IL-17A differed significantly across groups in the global Chi-square (χ^2^) test (*p* = 0.018) and showed a significant monotonic increase with disease severity in the χ^2^ test for trend (*p* = 0.005; Fig. 2c). After age adjustment by logistic regression, the overall group effect remained significant (*p* = 0.011) and the age-adjusted trend approached significance (*p* = 0.056). Compared to healthy controls, the adjusted odds ratio (OR) for detectability was 2.22 (95% CI [0.18–26.78]) for gingivitis and 13.93 (95% CI [1.01–192.12]) for periodontitis. IL-6 showed a similar pattern with higher detectability in diseased groups, reaching significance in the χ^2^ trend test (*p* = 0.036) but not in the global χ^2^ comparison (*p* = 0.107). Age-adjusted trend approached significance (*p* = 0.070) with OR 1.98 (95% CI 0.40–9.82) for gingivitis vs. healthy and 6.57 (95% CI [0.88–49.35]) for periodontitis vs. healthy. While TGF-β1 did not reach statistical significance, its detection frequency was roughly twofold higher in periodontitis compared to health and gingivitis. Other analyzed cytokines (IL-4, IL-2, IL-10, IL-12p70, IFN-γ and TNF-α) were not significantly affected among the groups.

### Characterization of circulating CD5^+^ B cells among study groups

**Fig. 3:**
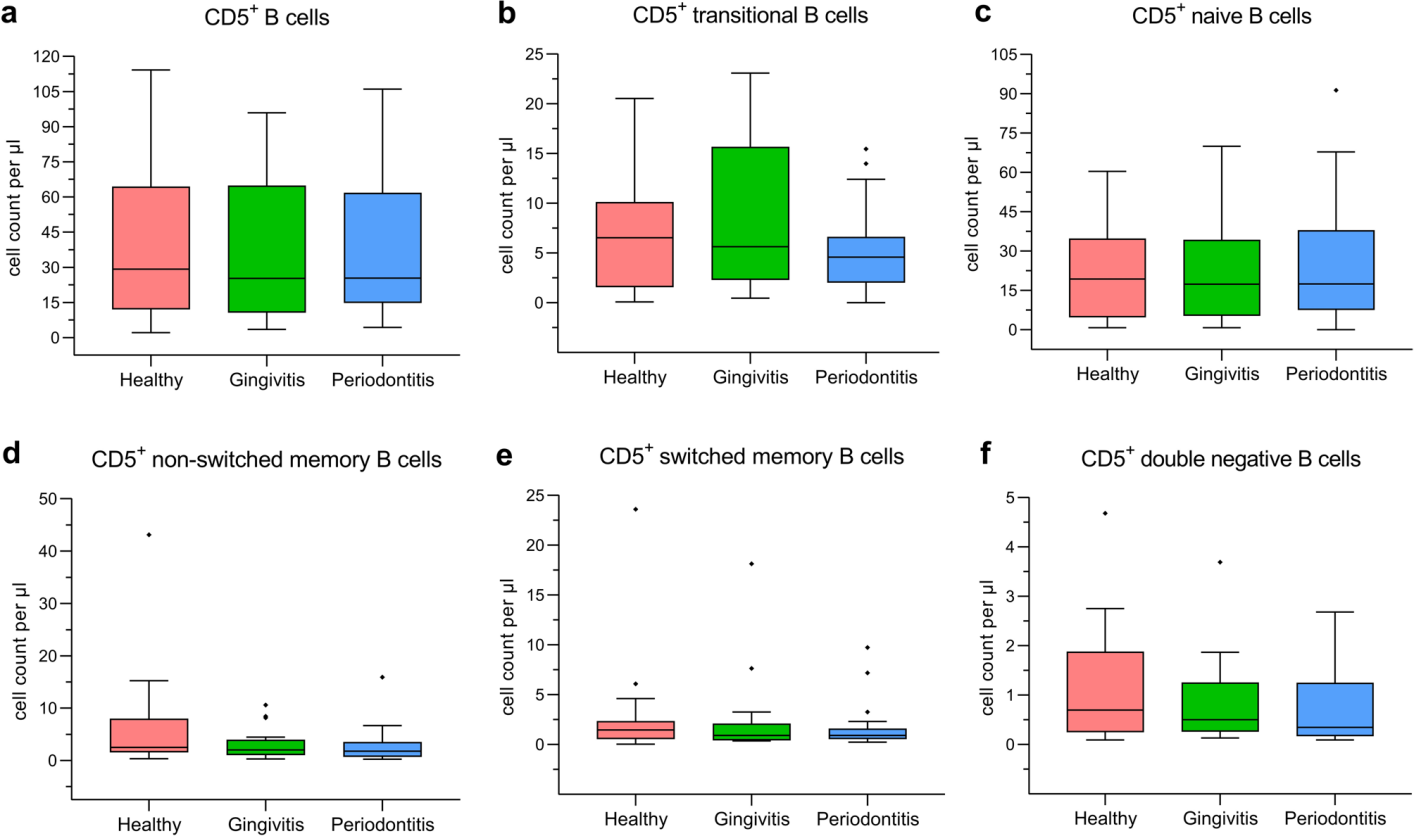
Flow cytometry analysis of the CD5^+^ B cell development. For each study group, cell count per µl blood was plotted for (**a**) all CD5^+^, (**b**) transitional, (**c**) naive, (**d**) non-switched memory, (**e**) switched memory and (**f**) double negative CD5^+^ B cells. The box-and-whisker plots indicate the median value (horizontal line), the interquartile range (IQR as bottom and top of the box) and the whiskers extending to the extreme values within 1.5xIQR. Points beyond the whiskers are outliers as defined by the Tukey method. N = 20 in all groups.

Circulating CD5^+^ B cells were characterized by flow cytometry to classify developmental stages. Comparison of absolute CD5^+^ B cell counts showed no significant differences between groups (Fig. 3). The ratios of all B lymphocytes, all CD5^+^ B cells as well as transitional and naive CD5^+^ subtypes were distributed evenly among the study groups (Table 1). The fractions of memory cells (non-switched, switched and double negative) were reduced by 41–53% in diseased groups, although not significantly different.

**Table 1:** Distribution of CD5^+^ B cells among study groups.

Cell type	Ratio [%]			Kruskal–Wallis
	Healthy	Gingivitis	Periodontitis	*p* value
B cells of all lymphocytes	10.5 ± 4.5	10.8 ± 3.9	11.3 ± 6.0	0.9724
CD5^+^ of all lymphocytes	2.3 ± 1.6	2.4 ± 2.1	2.3 ± 1.6	0.9679
CD5^+^ of all B cells	21.8 ± 12.3	19.8 ± 12.5	20.5 ± 9.3	0.8696
transitional CD5^+^ B Cells of all B cells	3.7 ± 2.4	4.4 ± 3.1	3.6 ± 3.3	0.5302
naive CD5^+^ B Cells of all B cells	11.7 ± 7.0	12.3 ± 9.4	13.1 ± . 8.0	0.8116
non-switched memory CD5^+^ B cells of all B cells	3.8 ± 5.4	1.6 ± 1.1	1.4 ± 1.0	0.1579
switched memory CD5^+^ B cells of all B cells	1.7 ± 2.3	1.0 ± 1.1	0.8 ± 0.5	0.8082
double negative CD5^+^ B cells of all B cells	0.7 ± 1.0	0.4 ± 0.3	0.4 ± 0.3	0.2492

Ratio comparison of peripheral blood B lymphocytes that express different cell surface markers corresponding to various developmental stages of CD5^+^ B cells. Mean values ± S.D. with n = 20 in each group are presented. Significance was determined using Kruskal–Wallis test.

Regression analysis found no statistical association between periodontitis and transitional (*p* = 0.174), naive (*p* = 0.176), non-switched (*p* = 0.962) or switched memory cells (*p* = 0.363). However, the fraction of double negative B cells might be potentially linked to periodontitis (OR = 0.023 with 95% CI [0.0–1.26], *p* = 0.065).

### Periodontal status of gingival crevicular fluid (GCF) collection sites

Clinical parameters at the sites of GCF collection were additionally evaluated (Supplementary Table S3). In the periodontitis group, the sampled sites displayed a mean PD of 4.54 ± 0.40 mm, with 79% of sites exceeding 4 mm and 18% of sites ≥ 6 mm. The corresponding pCAL averaged 1.88 ± 0.22 mm, with 21% of sites exceeding 3 mm and 11% ≥ 5 mm. In contrast, healthy and gingivitis group showed shallow PDs (2.20–2.4 mm) and no attachment loss. Plaque prevalence at the sampled sites in gingivitis (72%) was comparable to periodontitis (70%), while healthy participants presented approximately 30% less plaque. Bleeding on probing increased gradually from 5% in health to 55% in gingivitis and reached 78% in periodontitis.

### Taxonomic composition of the subgingival microbiome

**Fig. 4:**
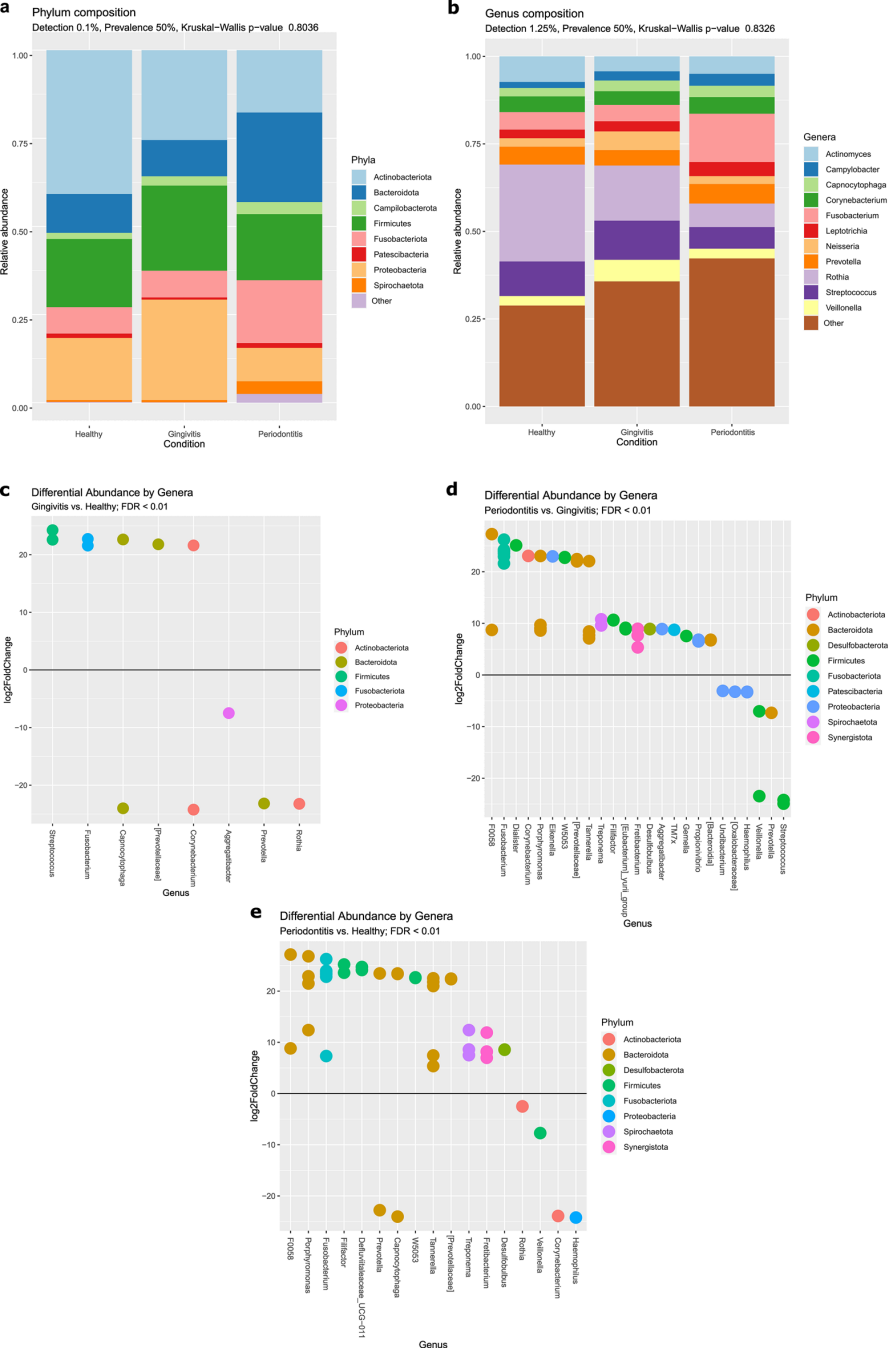
Taxonomic composition of oral microbiome in healthy and diseased groups. Stacked bar charts represent the cumulative relative abundance of bacterial (**a**) phyla and (**b**) genera across analyzed groups. Only taxa present in at least 50% of samples (prevalence threshold) are shown. The detection threshold was set at a relative abundance of 0.1% for phyla and 1.25% for genera. Less abundant or unclassified taxa are noted as Other. The Kruskal–Wallis test indicated no significant differences in the overall bacterial composition between the groups. Differential abundance analysis was performed to identify significantly enriched genera in (**c**) gingivitis (positive log2 fold change values) compared to healthy samples (negative log2 fold change), (**d**) periodontitis vs. gingivitis and (**e**) periodontitis vs. healthy. Each dot corresponds to a bacterial genus, color-coded according to its phylum as shown in the legend on the right. Significance was determined using the Wald test and the Benjamin-Hochberg correction with DESeq2. Genera with a FDR < 0.01 were included, indicating strong statistical significance in their differential abundance with n = 20 in healthy and periodontitis and n = 19 in gingivitis group.

At the phylum level (Fig. 4a), 16 s rRNA sequencing revealed a 50% reduction in Actinobacteriota and Proteobacteria in periodontitis samples compared to healthy controls (41% to 18% and 18% to 9%, respectively). In contrast, Bacteroidota, Fusobacteriota and Spirochaetota increased notably in periodontitis (11% to 25%, 7% to 18% and 1% to 4%). The Firmicutes to Bacteroidetes ratio decreased with inflammation (Supplementary Fig. S1). At the genus level, *Rothia* and *Streptococcus* were dominant in healthy samples but declined with disease progression (Fig. 4b). *Campylobacter*, *Capnocytophaga*, *Corynebacterium*, *Leptotrichia*, *Prevotella*, *Neisseria* and *Veillonella* were distributed evenly across groups*.* Less abundant genera (Other) increased with inflammation (29% vs. 36% vs. 42% in healthy vs. gingivitis. vs. periodontitis). Core microbiome data are provided in the Supplementary Material (Supplementary Fig. S2).

Differential analysis confirmed significant microbial shifts. Healthy microbiomes were enriched with *Rothia* and *Aggregatibacter*, while gingivitis samples had higher levels of *Streptococcus* and *Fusobacterium* (Fig. 4c). As the disease progressed from gingivitis to periodontitis, significant increases in Bacteriota, Fusobacteriota, Synergistota, Spirochaeta and Desulfobacterota could be observed (Fig. 4d). The prominent accumulation of Bacteroidota in periodontitis was especially profound in comparison to healthy group (Fig. 4e). Diverse members of *Fusobacterium*, *Speriochaetota* and *Synergistota* were highly enriched in diseased simples, while *Haemophilus*, *Corynebacterium*, *Veilonella* and *Rothia* significantly prevailed in healthy subjects.

### Community composition and functional pathway differences in the subgingival microbiome

The subgingival microbiome in periodontitis showed higher bacterial diversity but more individual variation compared to healthy or gingivitis states (Supplementary Fig. S3). Periodontitis was also marked by a loss of aerobic, gram-positive and biofilm-forming bacteria combined with an increase in anaerobic, gram-negative and pathogenic species (Supplementary Fig. S4). Functional shifts included elevated glycan, lipid and amino acid metabolism (Supplementary Fig. S5).

### Correlation analysis between clinical data and microbiome

Clinical data, measured cytokines levels and CD5^+^ B cell subsets were tested for a correlation with microbiome genera. A positive correlation was identified in gingivitis samples between CXCL8 (IL-8) and genus *Megasphaera* (R^2^ 0.859, *p* = 0.0014). The other two groups (healthy and periodontitis) showed no significant correlations with any of the clinical or immunological parameters.

### Identification of potential microbial biomarkers of periodontitis

**Fig. 5:**
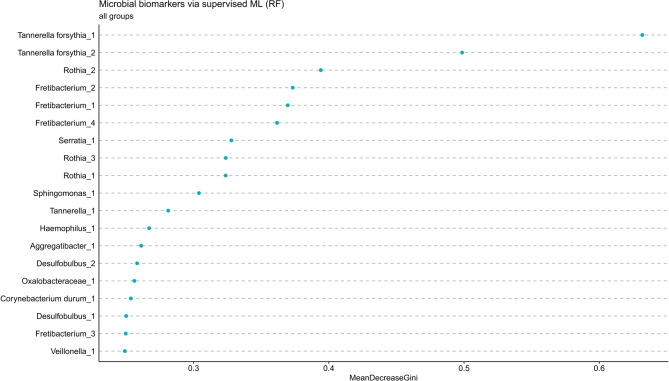
Potential biomarkers predicting transition from gingivitis to periodontitis. Random forest (RF) was used as a model of supervised machine learning (ML) to identify key microbial taxa that distinguish between healthy and diseased microbiome. To assess their importance, the Mean Decrease in Gini impurity was calculated for each taxon. This reflects how much a variable contributes to reducing node impurity in the decision trees of the model. Higher values indicate a greater impact on classification accuracy.

Random forest model was used to identify bacterial species that could predict the transition from healthy state to periodontitis (Fig. 5). This approach suggested that the most important predictors were two strains of *Tannerella forsythia*, three members of *Fretibacterium* and a *Rothia* genus.

Furthermore, generalized linear model was applied to test for associations between all acquired microbiome results (core microbiome, differential abundance, random forest) and specific genera. All 26 evaluated taxa explained the differences between the study groups, except for a member of the *Saccharimonadales*. *Cutibacterium* was linked specifically to the gingivitis group. Genera associated with periodontitis samples included *Aggregatibacter*, *Porphyromonas*, *Selenomonas*, *Tannerella*, *Fusobacterium*, *Treponema*, *Dialister* and *Eikenella*.

### Effect size distribution among significant findings

**Fig. 6:**
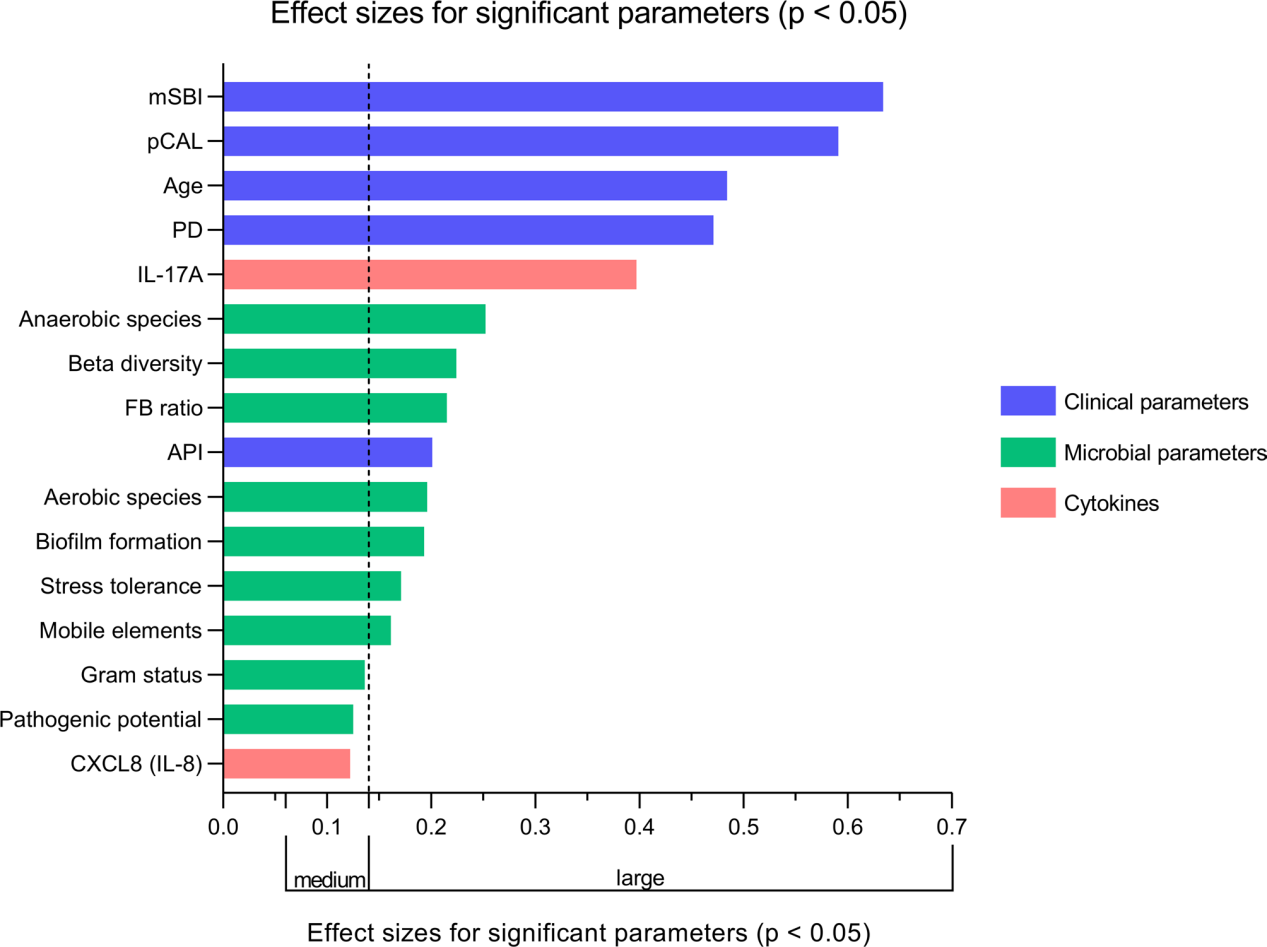
Effect sizes for parameters with significant group differences. Medium and large effect sizes (η^2^) derived from Kruskal–Wallis tests are shown for all continuous parameters that demonstrated significant differences between diagnostic groups (*p* < 0.05). Parameters are grouped by clinical, microbial or immunological (cytokines) category. Visualized thresholds for η^2^: medium ≥ 0.06, large ≥ 0.14. As the only significant binary variable, the IL-17A was analyzed by Chi-square test with Cramer’s *V* as the effect size (thresholds for Cramer’s *V*: medium ≥ 0.20, large ≥ 0.35; thresholds not visualized).

The magnitude of group differences was evaluated using η^2^ as effect size for all continuous parameters analyzed by Kruskal–Wallis test and Cramer’s *V* as effect size for all binary cytokine variables analyzed by Chi-square test. Figure 6 highlights the effect sizes of parameters that demonstrated significant differences across the groups (*p* < 0.05). A strong separation between diagnostic groups was indicated by mSBI followed by pCAL, age and PD (η^2^ > 0.45). Microbial characteristics such as oxygen utilization type (aerobic/anaerobic), beta diversity, FB ratio, biofilm formation, stress tolerance, mobile genetic elements, the clinical marker API and IL-17A also showed large effect sizes (0.14 < η^2^ < 0.45 and 0.35 < *V*, respectively). Moderate effects (0.06 < η^2^ < 0.14) were observed for gram status of microbes and their pathogenic potential as well as for IL-8.

The post hoc power analysis indicated that the study was sufficiently powered (> 80%) to detect meaningful differences between groups in all assessed clinical parameters as well as several key microbiome-related features (Supplementary Table S7). For IL-8 and IL-17A, the estimated power was moderate (70.4% and 78.5%, respectively). For remaining parameters, power estimates fell below 60%.

## Discussion

This study investigates potential biomarkers for predicting periodontal disease progression, focusing on circulating CD5^+^ B cells, salivary cytokines and subgingival microbiome. While CD5^+^ B cells are elevated in advanced inflammation^19,20^, their role in early stages remains unclear. Our findings reveal no significant association between analyzed CD5^+^ B cells and early disease progression, although memory cells tended to decrease with inflammation progress. Significantly elevated IL-8, IL-17A and TGF-β1 levels in saliva of periodontitis patients suggests their pivotal role in the onset of inflammation. Microbiome analysis further identified distinct shifts toward pathogenic profiles, with a positive correlation between IL-8 and *Megasphaera* in gingivitis, highlighting its potential as a biomarker for early disease dynamics.

The IL-17A, IL-8, IL-6, IL-1β and TGF-β1 play interconnected roles in inflammation, contributing to bone degradation and immune responses. Functional microbiome analysis revealed enhanced activity in bacterial glycan and LPS metabolism in diseased patients, correlating with increased presence of pathogenic genera like *Porphyromonas* (*P.*), *Tannerella* (*T.*), *Fusobacterium* (*F.*) and *Prevotella*, which are known for production of diverse virulence factors including LPS and peptidoglycan fragments^21–24^. Bacterial components typically trigger activation of pattern recognition receptors (PRRs) in immune and resident cells^25^, driving IL-1β and TGF-β1 secretion that are linked to bone loss^26–29^. The release of IL-1β stimulates epithelial cells for IL-6 and IL-8 production^30^. IL-8 is a potent neutrophil chemoattractant and can persist in the tissues for several weeks^31^. Migrated neutrophils release various collagenolytic enzymes and reactive oxygen species further contributing to tissue breakdown. IL-1β and IL-6 act synergistically with TGF-β1 to promote T-helper 17 (Th17) cells differentiation^32^, which is essential for mucosal defense. In our cohort, IL-8 concentrations were significantly increased in periodontitis cases, while IL-1β showed a distinct trend to higher levels. IL‑6 was also associated with disease progression when analyzed as detection frequency, revealing an increasing significant trend across groups. Similarly, IL-17A showed a significant rise in the number of detectable samples, whereas TGF-β1 (although not reaching statistical significance) was detected about twice as often in periodontitis as in healthy or gingivitis groups. This pattern likely reflects an early activation of the IL-1β/IL-8 axis and an emerging Th17-associated environment that is typically linked to IL-17 production^33,34^, consistent with prior studies associating IL-17 with inflamed periodontium^35–38^. Other analyzed cytokines remained stable, suggesting that early inflammation is primarily driven by IL-17A and IL-8 production.

In 2002, Berglundh et al*.*^14^ reported a threefold increase in CD5^+^ B cells in the blood of patients with advanced periodontitis compared to 15% in healthy individuals. Similarly, Sugawara et al*.*^16^ observed ca. 17% CD5^+^ B cells in advanced periodontitis vs. ~ 14% in healthy group. The link between systemic accumulation of CD5^+^ B cells and bone resorption is also evident in the case of rheumatoid arthritis, where severity of inflammation and bone degradation is eminently high. For example, Leandro et al*.*^39^ observed that in case of discontinued rituximab medication in RA patients, the proportion of CD5^+^ B cells in blood has risen from 33.7% to 78.7% within few months after therapy termination. A study by Engelmann et al*.*^17^ indicated a positive correlation between osteoclasts activity marker and CD5^+^ cells in blood of RA patients. In contrast, our data showed no changes in circulating CD5^+^ B cells, averaging 20% in both healthy and inflamed periodontium. We additionally examined developmental stages of CD5^+^ cells aiming to detect a potential shift in the cell development that would correlate with the disease progression. Transitional cells, representing immature B cells, as well as naive (mature) cells showed consistent levels, indicating a stable bone marrow output regardless of health status^40^. Upon antigen recognition, naive cells differentiate to plasma or memory cells, which are crucial for adaptive immunity. Non-switched memory cells (CD27^+^IgD^+^) provide rapid action upon re-exposure to antigens, while switched memory cells (CD27^+^IgD^−^) are linked to long-term immunity^41^. Double negative memory cells (CD27^−^IgD^−^) are implicated in autoimmunity, chronic inflammation and aging. However, this cell type remains poorly understood^42^. In our study, although not statistically significant, particularly non-switched and switched memory cells decreased by 50% in diseased patients, possibly due to migration to inflamed tissues^43^. Double negative cells may play a role in periodontitis, as indicated by our regression analysis, but statistical significance was not achieved likely due to the limited sample size. A larger cohort in future studies may help to clarify the association between this cell type and periodontitis. On the other hand, moderate periodontal disease may also lack sufficient inflammatory burden to induce systemic immune effects.

The inflammatory burden in periodontitis stems from an imbalance in oral microbiota. Pathogens such as *P. gingivalis*, *T. forsythia* and *Treponema (T.) denticola* play central roles in the pathogenesis and are known for inducing salivary IL-6, IL-8 and IL-17A secretion as shown in previous reports^44–48^. These bacterial genera were also highly prevalent in our periodontitis patients accompanied by significantly elevated levels of IL-8 as well as detection frequences of IL-6 and IL-17A, suggesting a potential link. Although we observed a positive correlation between IL-8 and *Megasphaera* in our gingivitis patients, other studies associate *Megasphaera* with healthy gums, demonstrating its ambiguous role^49,50^. Furthermore, the present study identified *Fretibacterium* as one of the most discriminative genera in the random forest model, supporting previous reports that suggested *Fretibacterium* as a novel biomarker for periodontitis screening^51–53^. Noteworthy, assignment of feature importance within random forest algorithm reflects statistical stability rather than established pathogenicity. Therefore, *T. forsythia*, *Fretibacterium* and *Rothia* showed more consistent abundance trends, capturing the gradual ecological shift from health to dysbiosis, while the absence of *P. gingivalis* and *T. denticola* among top features likely relates to the moderate disease severity in our cohort, where these pathogens were not uniformly dominant. Further taxonomic classification of periodontal flora displayed representative bacterial spectra depending on health status. Disease progression reflected a shift from gram-positive aerobic to gram-negative anaerobic bacteria, leading to dysbiosis and increased pathogenic risk. The demonstrated outcomes are consistent with the findings of several other studies that similarly profiled the oral microbiome^54–58^. In our study, participants were required to have at least 8 weeks since their last professional tooth cleaning to avoid the effects of recent prophylaxis on the subgingival environment. This timeframe is shorter than the traditional 6-month recall period, yet longitudinal data indicate that subgingival microbiota stabilizes within 6–12 weeks after cleaning^58–61^. While these cited studies focus on plaque, GCF originates from the same periodontal niche and reflects its microbial and inflammatory conditions^62^. Therefore, the GCF sampling window chosen for the present study fell within the biologically supported stabilization period.

From a clinical perspective, salivary IL-1β, IL-6, IL-17A and IL-8 have been proposed as markers for disease severity and therapy response^63–66^. Yet, systematic reviews highlight inconsistent evidence for IL-8^67,68^, while the diagnostic potential of IL-17A remains an active area of research^65,69–71^. In contrast, Ebersole et al. reported that salivary concentrations of IL-1β and IL-6 alone or in combination could distinguish health from gingivitis and periodontitis in the analyzed cohort^72^. Although our study was underpowered to detect significant effects for IL-1β and IL-6, both showed a clear upward trend. Given the multifactorial nature of periodontal inflammation and the considerable inter-individual variability in salivary cytokine levels, single biomarkers may not achieve sufficient diagnostic accuracy. Instead, combining complementary mediators such as IL-1β, IL-6, IL-17A and IL-8 into a multi-cytokine panel could provide a more accurate representation of the host response, improving sensitivity and specificity for early disease detection. Overall, saliva-derived cytokines more accurately reflect the local inflammation compared to serum cytokines, making them suitable for the evaluation of the therapeutic outcomes^73^. Emerging diagnostic approaches combine microbiological and salivary biomarkers to monitor the treatment success^74^. Accordingly, future research with larger cohorts should validate and optimize the multiplex approaches for clinical application and further investigate the relationship between IL-8 and *Megasphaera* in early inflammation, as both show promising potential as predictive markers for therapeutic outcomes.

Several limiting factors of our study should be acknowledged. The classification of periodontal diseases followed older guidelines^75^ rather than the newer radiographic-based system^76^. At the time of patient recruitment (2017–2019), the updated World Workshop classification had recently been introduced and had not yet become established in our routine diagnostic practice. Consequently, group classification was performed using the previously validated PD- and mSBI-based criteria. While the updated staging system provides a more nuanced clinical framework, our approach allows for a meaningful alignment with earlier studies and ensures comparability of findings within the existing literature. Moreover, clinical measures, microbial compositions and calculation of the effect sizes supported accurate group classification. Further concerns are related to the differences in participant age with younger individuals in healthy and gingivitis groups and older participants in the periodontitis group, which reflected the disease’s prevalence in seniors^2^, but may confound immune and microbial readouts. While age was included as a covariate in our regression analyses to statistically adjust for its influence, age matching was not achievable given the single-center recruitment and strict exclusion criteria limiting the number of recruitable age-matched participants. Additionally, the cytokine and microbiome analyses were performed in different matrices (saliva vs. GCF). Although previous work has successfully correlated salivary inflammatory mediators with subgingival microbiome profiles^77^ and demonstrated that saliva and GCF share a substantial proportion of host proteins and immune pathways^78^, direct cross-matrix interpretation of correlations observed in the present study should be considered as an exploratory, hypothesis-generating step linking localized dysbiosis to the broader inflammatory milieu. Furthermore, salivary cytokines and blood CD5^+^ B cells do not fully capture local periodontal immunological events, which would require sulcus fluid and gingival biopsies. The microbiome analysis was based on Operational Taxonomic Units (OTUs) rather than Amplicon Sequence Variants (ASVs), which may limit phylogenetic resolution and the ability to detect fine-scale microbial variation. However, due to the cross-sectional study design, longitudinal validation of microbiome alterations over time was not feasible. While this precludes causal inference, it does not compromise the robustness of the observed associations.

Given the cross-sectional design, the present data do not establish temporal precedence or predictive performance. Accordingly, the immunological and microbiological findings are interpreted as exploratory candidates requiring prospective validation. As for future directions, a focused study should enroll individuals with periodontal health or gingivitis and follow them for 12–18 months with examinations every 2–4 months, using pre-specified site-level progression endpoints consistent with the 2017 World Workshop case definition^76^. Sample size should be calculated power-based under conservative event-rate assumptions to avoid underpowered prognostic analyses^79^. Age-related confounding can be minimized through decade-based recruitment strata with analytical adjustment^80^. Biological comparability can be improved by matrix-matched, site-specific sampling. Model development and reporting should follow TRIPOD guidance^81,82^, with internal cross-validation and a single held-out participant set for final evaluation^83^, including discrimination as area under the curve (AUC), sensitivity/specificity, positive and negative predictive value (PPV/NPV) at a pre-specified threshold and calibration (intercept, slope, reliability plots)^84^. While this design suffices to establish predictive utility, functional assays may be used to support biological plausibility.

In summary, while circulating CD5^+^ B cells are of interest in periodontology due to their reported correlation with bone loss and elevation in severe periodontitis, our study was not able to distinguish between healthy, gingivitis and moderate periodontitis based on the ratio of CD5^+^ B cells in the blood. This suggests that early stages of periodontitis may lack sufficient systemic effects to induce CD5^+^ B cells proliferation as seen in more advanced disease. However, memory cells appeared reduced in the context of inflammation. In particular, the double negative CD5^+^ cell type warrants further investigation in larger cohorts. The assessed subgingival microbiome reflected patient health status, while salivary cytokine analysis indicated mild inflammation with elevated IL-8 levels and higher IL-17A detection rates in periodontitis. Additionally, an association between *Megasphaera* and salivary IL-8 was observed in gingivitis, which may have value for early-stage risk estimation, pending evaluation in larger, longitudinal, matrix-matched cohorts.

## Material and methods

### Study design and clinical evaluation

The study subjects were investigated from 2017 to 2019 and recruited from the Rostock University Medical Centre (Germany). All enrolled individuals gave their written informed consent. The ethics application has been approved by the ethics committee of the Rostock University Medical Center (A2014-0181). The study design and experiments were performed in accordance with the STROBE statement for observational studies and the Declaration of Helsinki. Exclusion criteria for subjects were smoking, chronic disease such as rheumatism, diabetes, HIV, antibiotic treatment within the last 6 months, periodontal treatment in the last two years, professional tooth cleaning within the last eight weeks, pregnancy or nursing.

Sixty participants were included in the study and classified into three groups according to their periodontal health status. Group sizes were balanced to allow meaningful comparisons of clinical, microbiological and immunological parameters: 20 subjects with moderate chronic periodontitis, 20 with gingivitis and 20 without evidence of periodontal disease as healthy controls. For B-cell analysis, blood samples were collected from all 60 participants. Due to the relocation of one participant after clinical evaluation and blood collection, saliva and crevicular fluid samples were missing in the gingivitis group resulting in 19 subjects available for cytokine and microbiome analysis.

Classification was based on probing depth (PD) and modified sulcus bleeding index (mSBI)^75^. For PD measurement, a full-mouth periodontal examination was performed using a UNC-15 periodontal probe at 6 sites per tooth, excluding third molars unless they were in the position of the second molars. Bleeding on probing was assessed in interdental areas using a dichotomous index (yes/no decision) at up to 28 sites per patient. The criteria for healthy subjects included a PD up to 3 mm at all sites, with the exception of max. 4 sites showing PD < 4 mm and an mSBI ≤ 10%. Gingivitis patients met the same PD criteria with mSBI > 10% indicating gingival inflammation. Periodontitis group required deeper pockets with PD ≥ 4 mm at a minimum of 4 sites and an mSBI > 10%.

Additionally, the approximal plaque index (API) and a proxy clinical attachment level (pCAL) were recorded in all patients for descriptive purposes and did not influence periodontal classification, which was based exclusively on PD and mSBI. To evaluate the API, a staining agent was applied to visualize plaque and its presence was assessed on the oral surfaces in quadrants 1 and 3 and on vestibular surfaces in quadrants 2 and 4 using a dichotomus scoring system (presence/absence of plaque). The pCAL was recorded full-mouth at six sites per tooth and calculated uniformly for all groups as pCAL = (PD–3 mm) + recession. The subtraction of 3 mm reflected the average physiological distance between the gingival margin and the cemento-enamel junction (CEJ) typically observed in healthy periodontium. This baseline correction was applied to approximate relative attachment loss in the absence of radiographs and CEJ-referenced attachment measurements in order to avoid overestimation of CAL in shallow sites. For sites with PD < 3 mm, pCAL was set to 0 mm. The pCAL measure was provided for illustrative purposes only and is not a validated CEJ-based CAL assessment.

### Cytokine profiling

For the cytokine determination, approximately 1 ml of saliva was collected from the patients by passive drooling into a sterile 1.5 ml microcentrifuge tubes. Subjects were asked to avoid eating, drinking and oral hygiene procedures for at least 1 h prior to sampling. Immediately after collection, samples were kept on ice and subsequently transferred to − 80 °C freezer until further use. On the day of analysis, samples were centrifuged at 3000 rpm for 15 min at 4 °C to remove cellular debris. The clarified supernatant was further processed for the cytokines detection using the 13-plex LEGENDPlex Human Essential Immune Response Panel (BioLegend, USA) according to the manufacturer´s instructions. The panel included detection of IL-4, IL-6, IL-2, IL-10, chemokine (C-X-C motif) ligand 10 (CXCL10, interferon-gamma induced protein 10 kD (IP-10)), interferon-gamma (IFN-γ), IL-1β, IL-20p70, TNF-α, CXCL8 (IL-8), CC-chemokine ligand 2 (CCL2, monocyte chemotactic protein 1 (MCP-1)), free active tumor growth factor- 1 beta (TGF-β1) and IL-17A. Stained cytokines were measured using FACS Verse (BD Biosciences, USA). The flow cytometry data were analyzed with the LEGENDplex™ Data Analysis Software Suite. The assay performance parameters such as lower limits of detection (LOD), upper limits of quantification (ULOQ) and intra-assay coefficients of variation (CVs; based on replicate median fluorescence intensity values) are reported in the Supplementary Table S8.

### B cells subset analysis by flow cytometry

Peripheral venous blood (7.5 ml) was collected into EDTA-coated S-Monovette® tubes (Sarstedt, Germany) by venipuncture. Tubes were kept at 4 °C and transported to the laboratory. Samples were processed within 2 h of collection. For isolation of peripheral blood mononuclear cells (PBMCs), 7 mL EDTA blood were diluted with 14 ml phosphate-buffered saline (PBS; pH 7.4) and carefully layered onto 15 ml Ficoll-Paque Plus (Cytiva, USA) in 50 ml tubes. Density separation was performed at 400 × g for 30 min at room temperature (RT) without brake. The mononuclear layer (~ 5–7 ml) was collected and washed in 20 ml autoMACS® Running Buffer (Miltenyi Biotec, Germany) by centrifugation at 500 × g for 7 min. Cells were resuspended in 10 ml autoMACS® Running Buffer, pelleted (300 × g, 7 min), resuspended in freezing medium at 2 × 10^6^ cells/ml and aliquoted (0.5 ml) into labeled cryovials. The freezing medium consisted of fetal calf serum (Biochrome, Germany) with 10% dimethyl sulfoxide (Roth, Germany). Cryovials were cooled at approx. − 1 °C/min rate using a freezing container (CoolCell, Biozym, Germany) in a − 80 °C freezer for ~ 24 h and then transferred to the vapor phase of liquid nitrogen (− 196 °C) for storage.

Prior to flow cytometry analysis, cryovials were thawed rapidly in a 37 °C water bath. Immediately after thawing, 1 ml of the cell suspension was diluted into 14 ml autoMACS® Running Buffer and centrifuged at 200 × g, 7 min. The pellet was resuspended in staining buffer (PBS with 0.5% BSA and 0.1% NaN_3_). Unless stated otherwise, cells were kept on ice and in the dark. For staining, 1 × 10^6^ cells were dispensed per tube, pelleted (850 × g, 7 min, 4 °C) and resuspended in 1 m staining buffer. Cells were stained with the viability dye eFluor 506 L/D (Invitrogen, USA; 1 µl per 1 ml of cell suspension; 30 min incubation at RT) and washed (850 × g, 7 min, 4 °C). Pellets were then resuspended in 50 µl autoMACS® Running Buffer, incubated with 5 µl Fc-block reagent (Human TrueStain FcX, Biolegend, USA) for 5 min to reduce nonspecific binding and stained with the B-cell panel for 15 min. Classification into lymphocytes, B cells and the individual developmental stages of CD5^+^ B cells was performed using surface marker staining with antibodies acquired from Biologend (USA) at the concentrations recommended by the manufacturer: CD45:PerCP antibody (clone HI30), IgD:FITC (clone: IA6-2), CD38:PerCP-Cy5.5 (clone HIT2), CD24:PE-Cy7 (clone ML5), CD27:APC (clone O323), CD19:BV785 (clone HIB19), CD5:BV421 (clone UCHT2). Samples were pelleted (850 × g, 7 min, 4 °C), resuspended in 500 µl autoMACS® Running Buffer and acquired on FACSAria IIIu (BD Biosciences, USA) with ≥ 1 × 10^5^ events per sample. Compensation was calculated using single-stained BD CompBeads (BD Biosciences, USA). Instrument quality control followed manufacturer guidance with daily BD FACSDiva CST Research Beads (BD Biosciences, USA) prior to acquisition. For sorting, drop-delay was verified using BD FACS Accudrop Beads (BD Biosciences, USA). Gating strategies were adopted from Engelmann et al*.* and can be found in the Supplementary Materials (MiFlowCyt-documentation) available at Rheumatology Online^17^. Vital lymphocytes were detected by a selection of CD45^+^ cells and vital B cells by the presence of CD19 marker. CD5^+^ B cells were further classified into naive [CD19^+^CD5^+^CD27^−^IgD^+^], transitional [CD19^+^CD5^+^CD27^−^IgD^+^CD38^++^CD24^++^], non-switched memory [CD19^+^CD5^+^CD27^+^IgD^+^], switched memory [CD19^+^CD5^+^CD27^+^IgD^−^] and double negative cells [CD19^+^CD5^+^CD27^−^IgD^−^].

### Sample processing for characterization of oral microbiome

For microbiome evaluation, gingival crevicular fluid (GCF) samples were taken with blunt, sterile paper points from the deepest periodontal pockets. For this purpose, the tooth was isolated with cotton rolls and gently air-dried. Supragingival plaque was gently removed with a scaler from the cervical area of the tooth to avoid contamination. The removed plaque material was not used for analysis and, therefore, was discarded. Four paper points with an ISO Size of 35 were inserted until resistance for 30 s each, then placed into an empty sterile 1.5 ml microtube and kept on ice until finishing the sampling. Visibly contaminated paper points (with blood or saliva) were discarded and replaced. Bleeding on probing (BOP) at the site of sampling was recorded as a binary measure (0/1). If bleeding occurred, sampling was performed only after visible bleeding had ceased. Collected samples were frozen at − 80 °C and stored until further use. The microbial DNA was isolated from GCF samples using ZymoBIOMICS DNA/RNA Miniprep Kit (Zymo Research, USA) following the manufacturer’s instruction on the DNA protocol. Before extraction, tubes were thawed on ice and processed immediately. Paper points were transferred into the kit’s lysis tubes as instructed. Eluates were stored at − 20 °C for ≤ 4 weeks. Preparation of 16S rRNA amplicon sequencing was performed according to previously published protocol^85^. A detailed description of 16S rRNA amplicon sequencing including primer sequences can be found in Supplementary Methods.

### Statistical analysis

Unless otherwise stated, both clinical and experimental data are presented as mean ± standard deviation (SD) or as median with interquartile range. Normality of continuous variables was assessed with the Shapiro–Wilk test within each diagnostic group. For most parameters, at least one group deviated from normality (Supplementary Table S9). Therefore, differences between groups were investigated using the Kruskal–Wallis test with Dunn’s post hoc correction. Probability levels considered as statistically significant were * *p* < 0.05, ** *p* < 0.01, *** *p* < 0.001 and **** *p* < 0.0001.

The majority of cytokines (IL-4, IL-2, IL-17A, IL-6, IL-10, IFN-γ, IL-12p70, TNF-α and TGF-β1) had less than 60% of values above the detection limit and were therefore analyzed as binary outcomes (detectable vs. non-detectable). For these variables, group differences were tested by Pearson’s Chi-square (χ^2^) tests (global and trend). Age-adjusted effects were evaluated with logistic regression including age as a covariate. Results are presented as odds ratios (ORs) with 95% confidence intervals (CI) and *p* values for the null hypothesis OR = 1. Cytokines with concentrations showing less than 5% of values below LOD (IL-1β, CXCL8/IL-8, CXCL10/IP-10 and CCL2/MCP-1) were compared among groups using the Kruskal–Wallis. For these continuous variables, values below LOD were imputed as LOD/2 and age-adjusted group effects were evaluated using rank-based ANCOVA models controlling for age.

To evaluate possible associations between the amount of CD5^+^ cells and the occurrence of periodontitis, a binary logistic regression model including several CD5^+^ cell types, controlled for age and gender, was fitted in a multiple approach. Results are presented as adjusted ORs with 95% CI and corresponding *p*-values. Gender distribution over three groups was tested by χ^2^ test.

Effect sizes and post hoc power analysis were calculated separately for continuous and binary outcomes. For continuous variables, effect sizes were derived from the H statistic (Kruskal–Wallis test) and summarized as eta-squared (η^2^) with conventional thresholds (small < 0.06, medium 0.06–0.14, large ≥ 0.14). Achieved post hoc power for these tests was obtained using the standard one-way ANOVA approximation to Kruskal–Wallis based on the observed effect magnitude, total sample size, number of groups and α = 0.05. For binary cytokine outcomes, across three groups, Cramér’s V (*V*) derived from the χ^2^ test was reported as the effect size. Interpretation followed common conventions for 2 × 3 tables (small < 0.20, medium 0.20–0.34, large ≥ 0.35). Achieved power for these χ^2^ tests was calculated using the observed effect magnitude, total sample size, degrees of freedom and α = 0.05.

Calculations and graph analysis were performed using GraphPad Prism 8.0.1, IBM® SPSS®, R-statistics program and Origin. Statistical analysis of microbiome data is described in Supplementary Methods.

## Supplementary Information

Below is the link to the electronic supplementary material.

Supplementary Material 1

## Data Availability

Data generated in this study are available upon reasonable request from the corresponding author. The Illumina sequencing data was deposited in the European Nucleotide Archive (ENA) database under the study accession number PRJEB82417 (https://www.ebi.ac.uk/ena/browser/view/PRJEB82417).
