# Supplementary material for: Potential biomarkers for early periodontal inflammation: investigating CD5+ B cells, salivary cytokines and oral microbiome
Source: Sci Rep. 2026 Feb 18;16:7192. doi: 10.1038/s41598-026-37044-6 (PMC12920879; doi:10.1038/s41598-026-37044-6)
Supplement: Supplementary file 1 — Supplementary Material 1 [file 41598_2026_37044_MOESM1_ESM.docx]

**Supplementary Material**

**Potential Biomarkers for Early Periodontal Inflammation: Investigating CD5^+^ B Cells, Salivary Cytokines and Oral Microbiome**

Elisabeth Clara Gottschalk ^1,*^, Oleksandra Chabanovska ^1,*, †^, Praveen Vasudevan ^1^, Israel Barrantes ^2^, Bernd Kreikemeyer ^3^, Wendy Bergmann-Ewert ^4^, Robby Engelmann ^4,5^, Brigitte Müller-Hilke ^4^, Hermann Lang ^1^

^1^ Department of Operative Dentistry and Periodontology, University Medical Center Rostock, Rostock, Germany

^2^ Research Group Translational Bioinformatics, Institute for Biostatistics and Informatics in Medicine and Ageing Research, University Medical Center Rostock, Rostock, Germany

^3^ Institute of Medical Microbiology, Virology and Hygiene, University Medical Center Rostock, Rostock, Germany

^4^ Core Facility for Cell Sorting and Cell Analysis, University Medical Center Rostock, Rostock, Germany

^5^ Clinic III (Hematology, Oncology and Palliative Medicine), Special Hematology Laboratory, Rostock University Medical School, Rostock, Germany

* These authors contributed equally

† Corresponding author: oleksandra.chabanovska@med.uni-rostock.de

**Results**

**Supplementary Table S1: Clinical characteristics of the patients classified into three groups according to periodontal health status.** Significance was determined using Kruskal-Wallis test followed by Dunn’s correction for multiple comparisons. Mean ± standard deviation (SD), median with interquartile ranges [IQR], mean *H* rank values and corresponding *p* values are presented. Abbreviations: PD = probing depth, pCAL= proxy attachment level, API = approximal plaque index, mSBI = modified sulcus bleeding index.

| **Groups** |  | **Clinical characteristics** | | | | |
| --- | --- | --- | --- | --- | --- | --- |
|  |  | **PD** | **pCAL** | **API** | **mSBI** | **Age** |
|  | **Unit** | mm | mm | % | % | years |
| **Healthy (H; n = 20)** | **Mean ± SD** | 1.75 ± 0.3 | 0.03 ± 0.06 | 33.35 ± 18.07 | 5.25 ± 2.29 | 32.5 ± 10.6 |
|  | **Median**  **[IQR]** | 1.75  [1.64 - 1.83] | 0.02  [0.01 - 0.03] | 36.00  [17.75 - 42.00] | 7.00  [4.00 - 7.00] | 29.0  [25.3 - 33.5] |
|  | ***H* rank** | 17.58 | 19.70 | 19.43 | 11.68 | 23.0 |
| **Gingivitis**  **(G; n = 20)** | **Mean ± SD** | 1.94 ± 0.33 | 0.05 ± 0.08 | 49.60 ± 19.74 | 24.55 ± 11.21 | 30.9 ± 7.9 |
|  | **Median**  **[IQR]** | 1.94  [1.78 - 2.24] | 0.02  [0.01 - 0.04] | 43.00  [39.00 - 63.25] | 23.00  [15.00 - 31.5] | 28.0  [25.5 - 34.3] |
|  | ***H* rank** | 27.23 | 22.53 | 32.88 | 35.23 | 20.8 |
| **Periodontitis (P; n = 20)** | **Mean ± SD** | 2.71 ± 0.80 | 0.79 ± 1.19 | 56.40 ± 22.13 | 35.60 ± 14.61 | 58.5 ± 15.5 |
|  | **Median**  **[IQR]** | 2.48  [2.23 - 2.94] | 0.34  [0.19 - 0.82] | 51.00  [43.75 - 73.5] | 38.50  [27.5 - 45.5] | 55.0  [47.3 - 73.0] |
|  | ***H* rank** | 46.7 | 49.28 | 39.20 | 44.6 | 47.8 |
| **p values** | ***H* vs. *G*** | 0.2416 | > 0.9999 | 0.0440 | < 0.0001 | > 0.9999 |
|  | ***G* vs. *P*** | 0.0013 | < 0.0001 | 0.7532 | 0.2638 | < 0.0001 |
|  | ***H* vs. *P*** | < 0.0001 | < 0.0001 | 0.001 | < 0.0001 | < 0.0001 |

**Supplementary Table S2: Mean percentage of sites** **per participant exceeding defined probing depth (PD) and proxy clinical attachment levels (pCAL) thresholds.** For all groups n = 20.

| **Groups** | **% of measured sites (Mean ± SD)** | | | |
| --- | --- | --- | --- | --- |
|  | **PD ≥ 4 mm** | **PD ≥ 6 mm** | **pCAL ≥ 3 mm** | **pCAL ≥ 5 mm** |
| **Healthy** | 0.94 ***±*** 1.01% | 0.0 ***±*** 0.0% | 0.06 ***±*** 0.29% | 0.0 ***±*** 0.0% |
| **Gingivitis** | 1.10 ***±*** 1.34% | 0.0 ***±*** 0.0% | 0.25 ***±*** 1.05% | 0.0 ***±*** 0.0% |
| **Periodontitis** | 19.13 ***±*** 17.43% | 4.19 ***±*** 9.86% | 10.97 ***±*** 22.08% | 2.72 ***±*** 8.30% |

**Supplementary Table S3: Local clinical characteristics of sites selected for gingival crevicular fluid (GCF) sampling.** For each participant, one representative site per quadrant (vestibular or palatal/lingual surface of a representative tooth) was examined for probing depth (PD) and proxy clinical attachment level (pCAL). Values are given as mean ± SD percentages that denote the mean proportion of sampled sites per participant fulfilling the respective criteria. For all groups n = 20.

| **Parameter** | **Healthy** | **Gingivitis** | **Periodontitis** |
| --- | --- | --- | --- |
| **PD (mm)** | **mean ± SD** | | |
| Q1 vestibular | 1.90 ***±*** 0.55 | 2.45 ***±*** 0.60 | 4.10 ***±*** 1.71 |
| Q2 palatal | 2.40 ***±*** 0.68 | 2.30 ***±*** 0.47 | 4.80 ***±*** 1.64 |
| Q3 lingual | 2.35 ***±*** 0.58 | 2.50 ***±*** 0.68 | 4.95 ***±*** 1.32 |
| Q4 vestibular | 2.15 ***±*** 0.59 | 2.30 ***±*** 0.57 | 4.30 ***±*** 1.34 |
| PD across quadrants | 2.20 ***±*** 0.23 | 2.39 ***±*** 0.10 | 4.54 ***±*** 0.40 |
| % sampled sites ≥ 4 mm  (per patient) | 0.00 ***±*** 0.00% | 0.00 ***±*** 0.00% | 78.75 ***±*** 21.88% |
| % sampled sites ≥ 6 mm  (per patient) | 0.00 ***±*** 0.00% | 0.00 ***±*** 0.00% | 17.50 ***±*** 31.52% |
| **pCAL (mm)** | **mean ± SD** | | |
| Q1 vestibular | 0.00 ***±*** 0.00 | 0.00 ***±*** 0.00 | 1.70 ***±*** 2.50 |
| Q2 palatal | 0.05 ***±*** 0.22 | 0.00 ***±*** 0.00 | 2.05 ***±*** 0.24 |
| Q3 lingual | 0.05 ***±*** 0.22 | 0.05 ***±*** 0.22 | 2.1 ***±*** 1.45 |
| Q4 vestibular | 0.00 ***±*** 0.00 | 0.00 ***±*** 0.00 | 1.70 ***±*** 1.68 |
| pCAL across quadrants | 0.02 ***±*** 0.03 | 0.01 ***±*** 0.03 | 1.88 ***±*** 0.22 |
| % sampled sites ≥ 3 mm  (per patient) | 0.00 ***±*** 0.00% | 0.00 ***±*** 0.00% | 21.25 ***±*** 30.65% |
| % sampled sites ≥ 5 mm  (per patient) | 0.00 ***±*** 0.00% | 0.00 ***±*** 0.00% | 11.25 ***±*** 28.65% |
| **% sampled sites with plaque**  (per patient) | 40.00 ***±*** 34.79% | 72.50 ***±*** 30.24% | 70.00 ***±*** 34.03% |
| **% sampled sites with BOP**  (per patient) | 5.00 ***±*** 15.39% | 55.00 ***±*** 35.91% | 77.50 ***±*** 25.52% |

**Supplementary Table S4: Descriptive statistics of salivary cytokines** **analyzed as continuous concentrations (pg/ml).** Results are shown as mean ± standard deviation (SD) and median with interquartile ranges [IQR] with n per group indicated in the header.

| **Cytokines** | **Healthy (n = 20)** | | **Gingivitis (n = 19)** | | **Periodontitis (n = 20)** | |
| --- | --- | --- | --- | --- | --- | --- |
|  | **Mean ± SD** | **Median**  **[IQR]** | **Mean ± SD** | **Median**  **[IQR]** | **Mean ± SD** | **Median**  **[IQR]** |
| **IL-1β** | 766.1 ± 2184.0 | 203.7  [139.0 - 524.8] | 345.3 ± 247.6 | 279.8  [171.2 - 546.2] | 1343.0 ± 1429.0 | 1048  [129.7 - 2331.0] |
| **CXCL8 (IL-8)** | 709.4 ± 901.3 | 454.5  [245.6 - 778.1] | 748.8 ± 679.1 | 551.6  [192.5 - 1191] | 2401.0 ± 2686.0 | 1190  [616.8 - 4302] |
| **CCL2 (MCP-1)** | 830.3 ± 454.2 | 721.2  [517.7 - 1233.0] | 951.5 ± 733.3 | 749.0  [449.1 - 1373.0] | 1255.0 ± 1015.0 | 805.0  [493.9 - 2268.0] |
| **CXCL10 (IP-10)** | 1659.0 ± 2486.0 | 936.7  [252.5 - 1430.0] | 721.3 ± 645.3 | 560.9  [145.9 - 1062.0] | 1306.0 ± 1579.0 | 612.0  [239.4 - 1594.0] |

**Supplementary Table S5: Unadjusted and age-adjusted statistical group comparisons for cytokines analyzed as continuous concentrations.** Cytokine levels were compared among healthy, gingivitis and periodontitis groups using the Kruskal-Wallis test (*H* statistic) with corresponding unadjusted *p* values. Age-adjusted *p* values were calculated using rank-based ANCOVA models with age as covariate.

| **Cytokines** | **H rank** | **Unadjusted p value** | **Age-adjusted p value**  **(rank-ANCOVA)** |
| --- | --- | --- | --- |
| **IL-1β** | 5.897 | 0.0524 | 0.0512 |
| **CXCL8 (IL-8)** | 8.836 | 0.0121 | 0.0101 |
| **CCL2 (MCP-1)** | 0.665 | 0.7170 | 0.7064 |
| **CXCL10 (IP-10)** | 0.900 | 0.6377 | 0.6492 |

**Supplementary Table S6: Unadjusted and age-adjusted group comparisons for binary cytokine detectability frequencies.** Shown are counts of detectable samples per group, *p*-values from unadjusted global Pearson’s Chi-square (χ²) test (global and trend) and corresponding age-adjusted *p*-values derived from binary logistic regression. Odds ratios (OR) and 95% confidence intervals (CI) compare gingivitis (G) and periodontitis (P) with the healthy (H) group. Total n = 20 in H and P, total n = 19 in G.

| **Cytokines** | **Detectable n (%)** | | | **Unadjusted p value** | | **Age-adjusted p value (logistic regression)** | | | |
| --- | --- | --- | --- | --- | --- | --- | --- | --- | --- |
|  | **Healthy** | **Gingivitis** | **Periodontitis** | χ² **(global)** | χ² **(trend)** | **global** | **trend** | **OR (95% CI) G vs. H** | **OR (95% CI) P vs*.* H** |
| **IL-4** | 1 (5.0%) | 3 (15.8%) | 2 (10.0%) | 0.5073 | 0.6009 | 0.5275 | 0.1867 | 3.41 (0.32 - 36.42) | 7.17 (0.31 - 164.56) |
| **IL-2** | 3 (15.0%) | 7 (36.8%) | 4 (20.0%) | 0.2980 | 0.7101 | 0.2562 | 0.3344 | 3.26 (0.70 - 15.27) | 1.77 (0.20 - 15.57) |
| **IL-17A** | 1 (5.0%) | 2 (10.5%) | 8 (40.0%) | 0.0184 | 0.0045 | 0.0105 | 0.0556 | 2.22 (0.18 - 26.78) | 13.93 (1.01 - 192.12) |
| **IL-6** | 3 (15.0%) | 5 (26.3%) | 9 (44.0%) | 0.1068 | 0.0362 | 0.1043 | 0.0746 | 1.98 (0.40 - 9.82) | 6.57 (0.88 - 49.35) |
| **IL-10** | 3 (15.0%) | 5 (26.3%) | 5 (25.0%) | 0.7311 | 0.4455 | 0.6303 | 0.1391 | 1.94 (0.39 - 9.69) | 5.27 (0.59 - 46.76) |
| **IFN-γ** | 3 (15.0%) | 2 (10.5%) | 5 (25.0%) | 0.2075 | 0.3849 | 0.4698 | 0.7646 | 0.67 (0.10 - 4.56) | 1.70 (0.20 - 14.72) |
| **IL-12p70** | 4 (20.0%) | 3 (15.8%) | 3 (15.0%) | 1.0000 | 0.6734 | 0.9045 | 0.7691 | 0.71 (0.13 - 3.73) | 1.99 (0.21 - 19.25) |
| **TNF-α** | 13 (65.0%) | 11 (57.9%) | 11 (55.0%) | 0.8033 | 0.5197 | 0.8021 | 0.7237 | 0.73 (0.20 - 2.67) | 0.82 (0.15 - 4.64) |
| **TGF-β1** | 5 (25.0%) | 6 (31.6%) | 10 (50.0%) | 0.2318 | 0.0987 | 0.234 | 0.3917 | 1.42 (0.35 - 5.80) | 2.13 (0.36 - 12.65) |

**Firmicutes - Bacteroidetes Ratio**

The highest median F/B ratio was detected in healthy samples followed by a slightly lower value in gingivitis group (3.56 [IQR 0.98 - 9.53] vs. 3.32 [IQR 1.62 - 5.27]). Although the difference in these two groups was not statistically significant, the shift towards Bacteroidetes-dominated community was highly prominent in periodontitis group with a median of 0.76 ([IQR 0.42 - 2.73]; *p* = 0.004 vs. gingivitis and *p* = 0.003 vs. healthy). Comparing mean values reflected this observation (decrease from 5.87 ± 6.05 to 3.76 ± 2.25 and further to 1.54 ± 1.69 in healthy to diseased groups; **Fig. S1**).


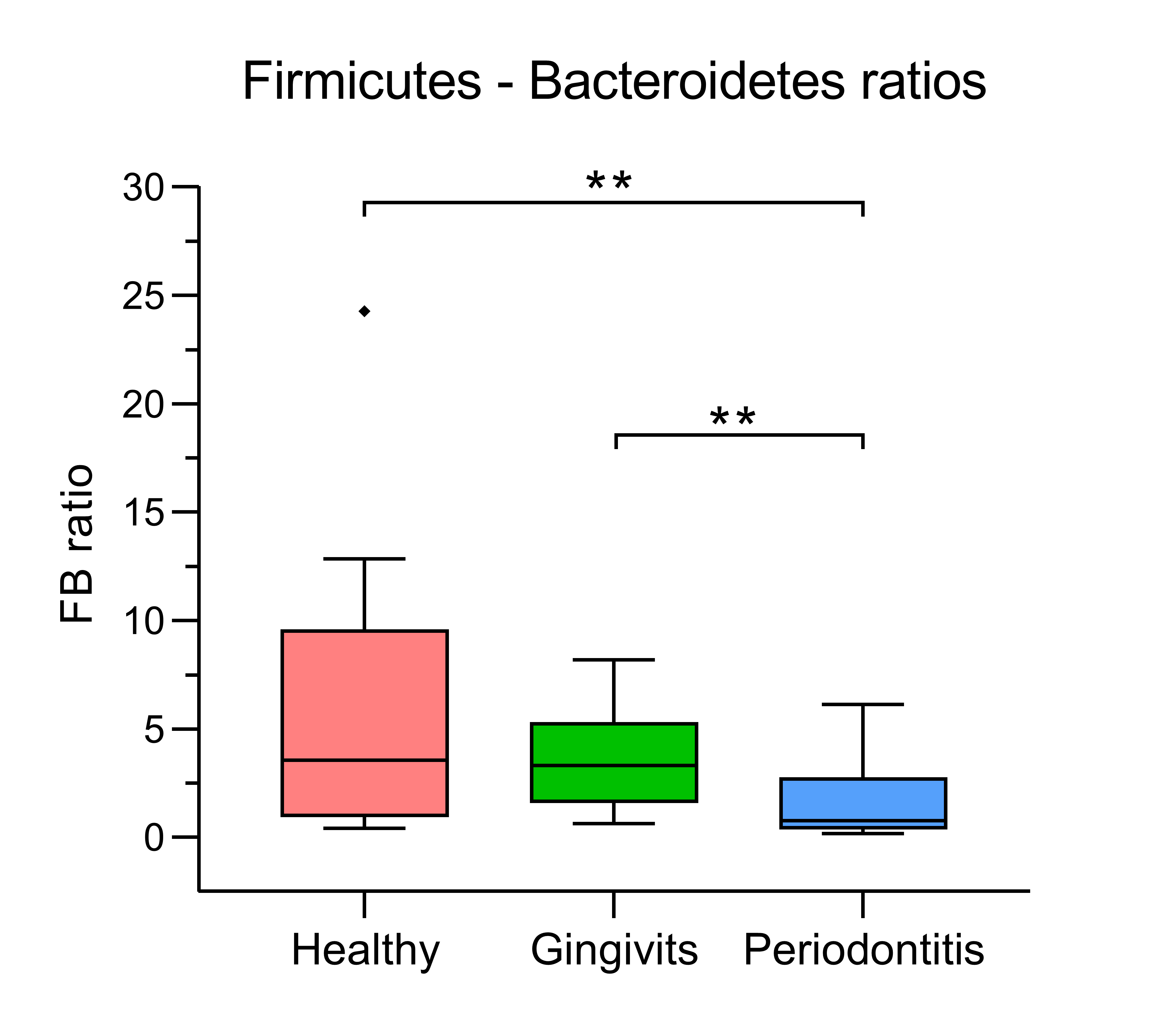


**Supplementary Figure S1: Firmicutes to Bacteroidetes ratio across study groups.** The box-and-whisker plots indicate the median value (horizontal line), the interquartile range (IQR as bottom and top of the box) and the whiskers extending to the extreme values within 1.5xIQR. Points beyond the whiskers are outliers as defined by the Tukey method. Significance was determined using Kruskal-Wallis test followed by Dunn’s correction for multiple comparisons (** p < 0.005) with n = 20 in healthy and periodontitis groups and n = 19 in gingivitis group.

**Core Microbiome**

In contrast to relative abundance analysis, which provides insights into proportions of most or least prevalent taxa, core microbiome data visualize consistently present taxa (prevalence of 80%) within a group, whereby excluding transient or rare bacteria. Thus, healthy core microbiome contained predominantly *Rothia* followed by *Streptococcus* and *Actinomyces* as the most consistently abundant genera (**Fig. S2a**). In gingivitis, *Streptococcus* gained noticeable growth (**Fig. S2b**). While *Rothia* and *Actinomyces* were still dominant genera, members of *Veillonella*, *Fusobacterium* and *Prevotella* demonstrated substantially increased presence compared to healthy controls. In periodontitis samples, the core microbiome consisted of highly abundant members of *Fusobacterium*, *Prevotella* and *Campylobacter* followed by *Corynebacterium*, *Streptococcus*, *Capnocytophaga* and *Leptotrichia* (**Fig. S2c**).


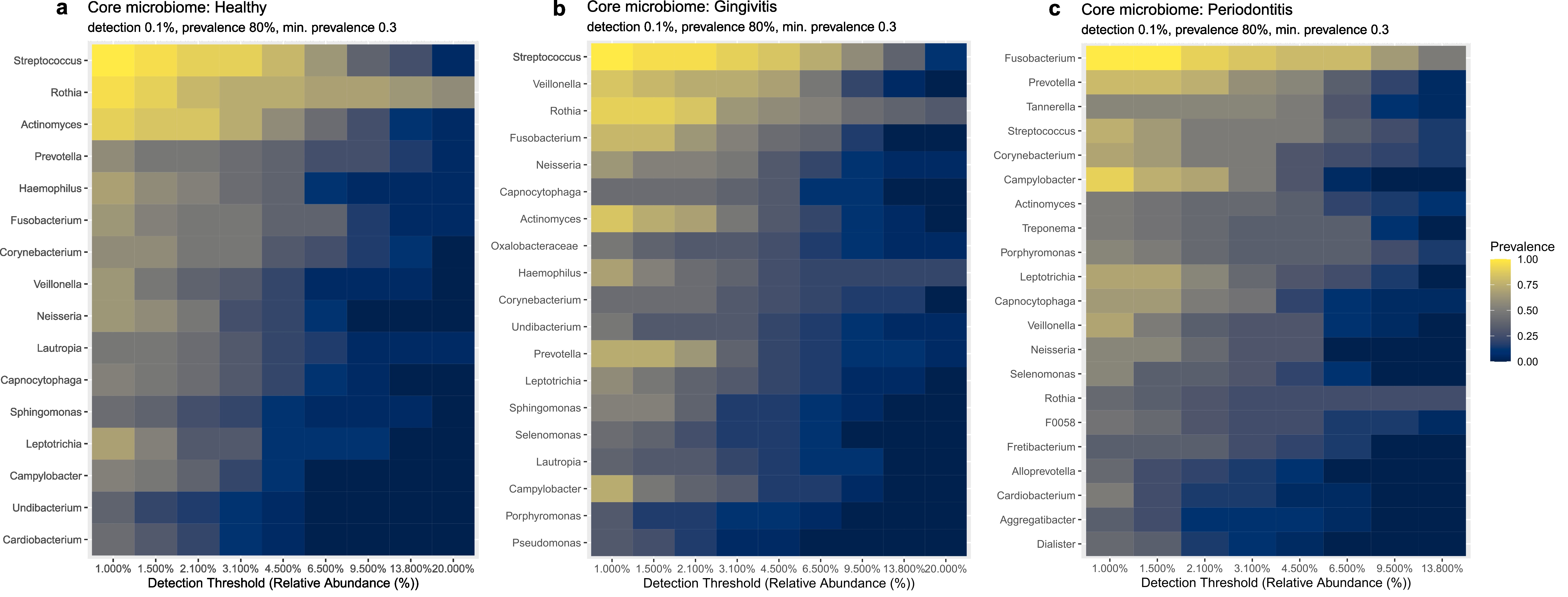


**Supplementary Figure S2: Core oral microbiome of individuals with healthy and diseased periodontal status.** Heatmap**s** illustrate prevalence of various bacterial genera (y-axis) across (**a**) healthy, (**b**) gingivitis and (**c**) periodontitis samples at different detection thresholds for relative abundance (x-axis). Prevalence at each abundance threshold is represented by a color gradient from dark blue (prevalence 0.0) to yellow (prevalence 1.00). The detection threshold at which a genus was considered to be present in a sample was set at 0.1%. Genera that appeared in less than 80% of the samples were excluded from the core microbiome (prevalence threshold). Genera identified with a minimum prevalence of 0.3 are shown in the heatmap.

**Community composition in the subgingival microbiome**


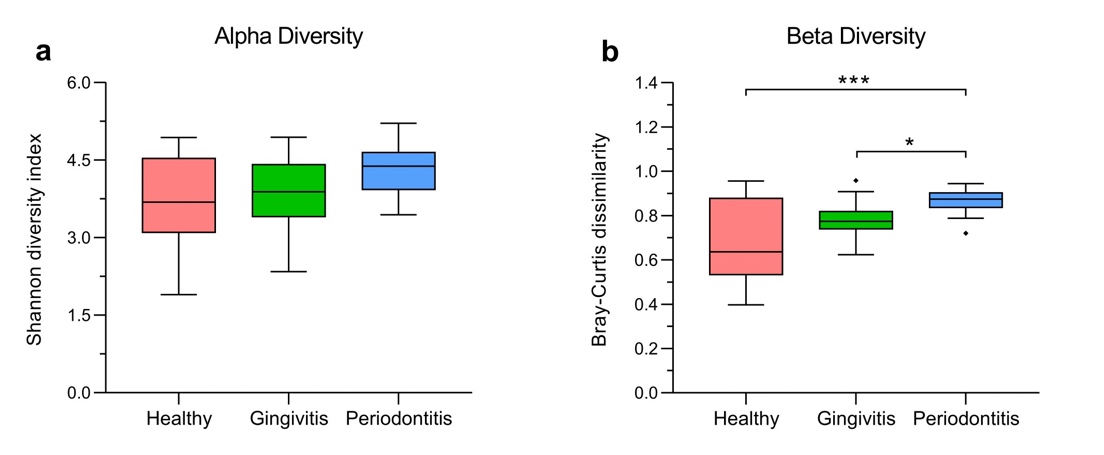
The richness (number) and evenness (distribution) of bacterial community within an individual sample was assessed by Shannon alpha diversity (**Fig. S3a**). A noticeable upward trend towards higher diversity was observed for the diseased groups. However, Bray-Curtis beta diversity indicated that the microbial composition under inflamed conditions highly varied between individuals (*p* = 0.004 vs. gingivitis and *p* = 0.003 vs. healthy), whereas bacterial community in healthy subjects appeared more stable (**Fig. S3b**).

**Supplementary Figure S3: Bacterial diversity analysis across study groups.** (**a**) Shannon index was calculated as a measure of alpha diversity to illustrate richness and distribution of species within an individual sample across the study groups. (**b**) The dissimilarity in microbial composition between individuals was illustrated by Bray-Curtis index as a measure of beta diversity. The box-and-whisker plots indicate the median value (horizontal line), the interquartile range (IQR as bottom and top of the box) and the whiskers extending to the extreme values within 1.5xIQR. Points beyond the whiskers are outliers as defined by the Tukey method. Significance was determined using Kruskal-Wallis test followed by Dunn’s correction for multiple comparisons (**p* < 0.05, *** *p* < 0.0005) with n = 20 in healthy and periodontitis groups and n = 19 in gingivitis group.

A significant loss of aerobic (*p* = 0.002), gram positive bacteria (*p* = 0.006) and biofilm-forming bacteria (*p* = 0.002) was observed in periodontitis group compared to healthy status (**Fig. S4a-c**). Accordingly, anaerobic (*p* = 0.0004), gram negative (*p* = 0.006) and potentially pathogenic species (*p* = 0.008) expanded significantly in the diseased patients (**Fig. S4d-f**), while the fraction of facultative anaerobic microbes did not differ significantly between groups (**Fig. S4g**). Species with high stress tolerance and bacteria containing mobile genetic elements (genetic transfer of antibiotic resistance or virulence factors) remained at similar proportions in comparison between healthy and periodontitis condition (**Fig. S4h-i**).

**Functional pathway differences in oral microbiome**

Differential abundance of KEGG (Kyoto Encyclopedia of Genes and Genomes) pathways highlighted the functional shift of microbial communities in periodontitis individuals. In comparison to healthy patients, a prominent enrichment of genes involved in glycan, sphingolipid and amino acid metabolism was detected in periodontitis samples (**Fig. S5a**). Comparison of periodontitis to gingivitis communities revealed a similar profile (**Fig. S5b**) with evident enrichment of lipopolysaccharide biosynthesis.


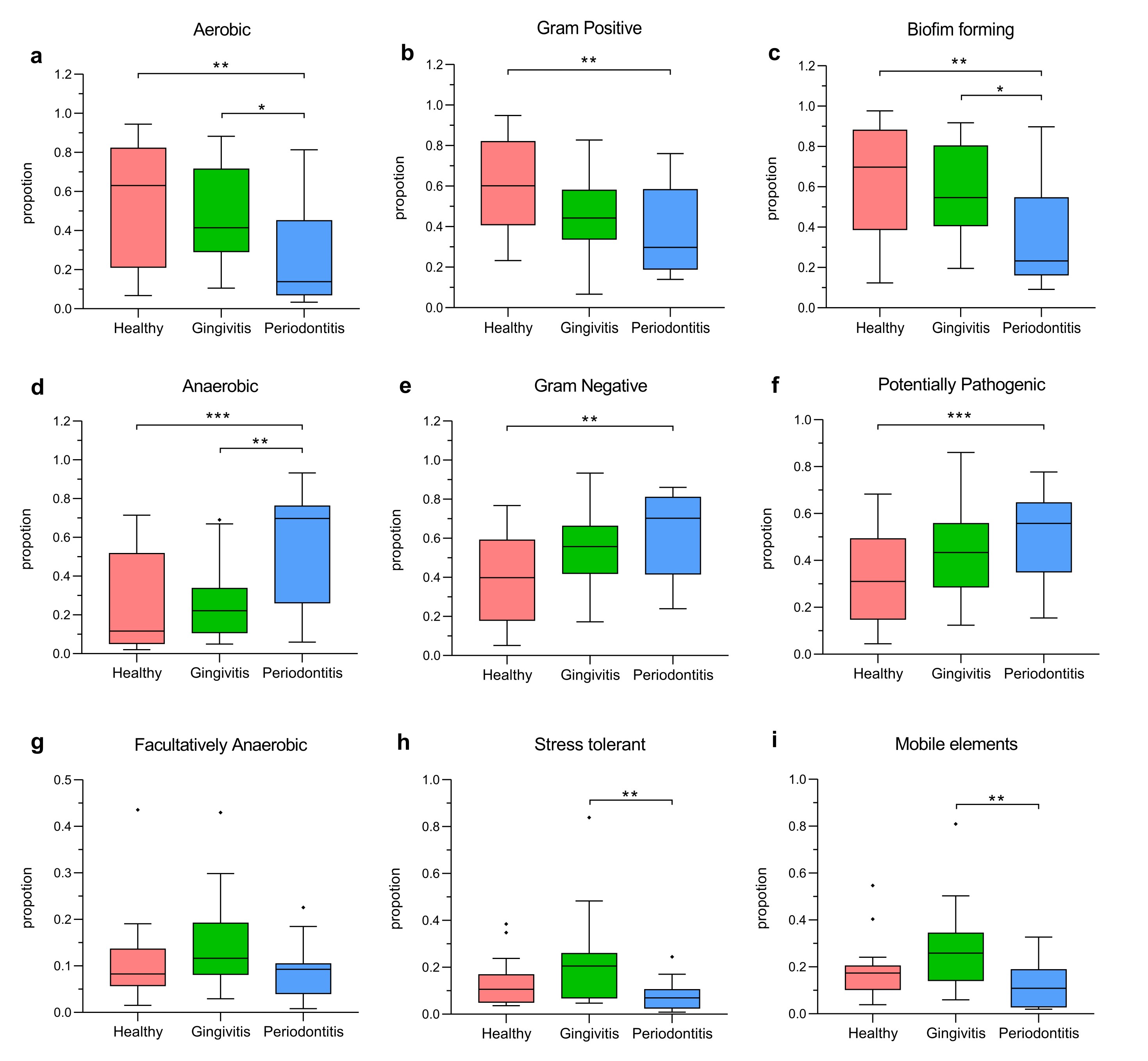


**Supplementary Figure S4: Community composition of oral microbiome in healthy and diseased groups.** Proportions of (**a**) aerobic, (**b**) gram positive, (**c**) biofilm forming, (**d**) anaerobic, (**e**) gram negative, (**f**) potentially pathogenic, (**g**) facultative anaerobic, (**h**) stress tolerant bacteria and bacteria containing (**i**) mobile genetic elements were evaluated in healthy, gingivitis and periodontitis samples. The box-and-whisker plots indicate the median value (horizontal line), the interquartile range (IQR as bottom and top of the box) and the whiskers extending to the extreme values within 1.5xIQR. Points beyond the whiskers are outliers as defined by the Tukey method. Significance was determined using Kruskal-Wallis test followed by Dunn’s correction for multiple comparisons (**p* < 0.05, ***p* < 0.005, *** *p* < 0.0005) with n = 20 in healthy and periodontitis groups and n = 19 in gingivitis group.





**Supplementary Figure S5: Functional pathway analysis of oral microbiome based on Kyoto Encyclopedia of Genes and Genomes (KEGG).** Differential abundance of KEGG pathways is presented for comparison between samples from individuals with (**a**) periodontitis vs. healthy controls and (**b**) periodontitis vs. gingivitis. The abundance of metabolic or functional pathways identified in microbiome varied between the two groups, with positive log2 (fold change) values indicating higher abundance in the former and negative values in the latter group.

**Supplementary Table S7: Summarized statistical results for effect sizes and power estimates for all assessed parameters.** For continuous parameters, Kruskal-Wallis results are shown: test statistic (*H*), *p* values, effect sizes (η^2^) and corresponding post hoc power estimates. For binary cytokine variables (detectable vs. not detectable), Chi-square results are reported instead: χ^2^, *p* values, Cramer’s *V* and post hoc power. Effect size thresholds for η^2^: medium ≥ 0.06, large ≥ 0.14; for Cramer’s *V* (df = 2): medium ≥ 0.20, large ≥ 0.35.

| **Category** | **Parameter** | **H rank**  **(χ^2^ for binary)** | **p value** | **Effect size η^2^**  **(V for binary)** | **Estimated post-hoc Power** | |
| --- | --- | --- | --- | --- | --- | --- |
| **Clinical data** | Age | 29.583 | < 0.0001 | 0.484 | 1.000 | High (>80%) |
| **Clinical data** | API | 13.435 | 0.001 | 0.201 | 0.933 | High (>80%) |
| **Clinical data** | pCAL | 35.678 | < 0.0001 | 0.591 | 1.000 | High (>80%) |
| **Clinical data** | mSBI | 38.135 | < 0.0001 | 0.634 | 1.000 | High (>80%) |
| **Clinical data** | PD | 28.875 | < 0.0001 | 0.472 | 1.000 | High (>80%) |
| **Cytokines**  **(binary)** | IL-4 | 1.243 | 0.5073 | 0.145 | 0.155 | Very Low (<40%) |
| **Cytokines**  **(binary)** | IL-2 | 2.801 | 0.2980 | 0.218 | 0.302 | Very Low (<40%) |
| **Cytokines**  **(binary)** | IL-17A | 9.294 | 0.0184 | 0.397 | 0.785 | Moderate (60-80%) |
| **Cytokines**  **(binary)** | IL-6 | 4.473 | 0.1068 | 0.275 | 0.458 | Low (40-60%) |
| **Cytokines**  **(binary)** | IL-10 | 0.881 | 0.7311 | 0.122 | 0.122 | Very Low (<40%) |
| **Cytokines**  **(binary)** | IFN-γ | 3.435 | 0.2075 | 0.23 | 0.363 | Very Low (<40%) |
| **Cytokines**  **(binary)** | IL-12p70 | 0.204 | 1.0000 | 0.059 | 0.066 | Very Low (<40%) |
| **Cytokines**  **(binary)** | TGF-β1 | 2.923 | 0.2318 | 0.223 | 0.314 | Very Low (<40%) |
| **Cytokines**  **(binary)** | TNF-α | 0.438 | 0.8033 | 0.086 | 0.084 | Very Low (<40%) |
| **Cytokines** | IL-1β | 5.897 | 0.0524 | 0.070 | 0.432 | Low (40-60%) |
| **Cytokines** | CXCL8 (IL-8) | 8.836 | 0.0121 | 0.122 | 0.704 | Moderate (60-80%) |
| **Cytokines** | CCL2 (MCP-1) | 0.665 | 0.7170 | 0.000 | 0.000 | Very Low (<40%) |
| **Cytokines** | CXCL10 (IP-10) | 0.900 | 0.6377 | 0.000 | 0.000 | Very Low (<40%) |
| **B cells** | B cells | 0.273 | 0.873 | 0.000 | 0.050 | Very Low (<40%) |
| **B cells** | CD5^+^ B cells | 0.007 | 0.997 | 0.000 | 0.050 | Very Low (<40%) |
| **B cells** | CD5^+^ double negative B cells | 1.535 | 0.464 | 0.000 | 0.050 | Very Low (<40%) |
| **B cells** | CD5^+^ naive B cells | 0.250 | 0.882 | 0.000 | 0.050 | Very Low (<40%) |
| **B cells** | CD5^+^ non-switched memory B cells | 2.125 | 0.346 | 0.002 | 0.060 | Very Low (<40%) |
| **B cells** | CD5^+^ switched memory B cells | 0.255 | 0.880 | 0.000 | 0.050 | Very Low (<40%) |
| **B cells** | CD5^+^ transitory B cells | 1.594 | 0.451 | 0.000 | 0.050 | Very Low (<40%) |
| **Microbiome** | Aerobic | 12.991 | 0.002 | 0.196 | 0.921 | High (>80%) |
| **Microbiome** | Anaerobic | 16.126 | 0.000 | 0.252 | 0.980 | High (>80%) |
| **Microbiome** | Beta diversity | 14.571 | 0.001 | 0.225 | 0.959 | High (>80%) |
| **Microbiome** | Biofim-forming | 12.817 | 0.002 | 0.193 | 0.916 | High (>80%) |
| **Microbiome** | Facultatively Anaerobic | 5.602 | 0.061 | 0.064 | 0.401 | Low (40-60%) |
| **Microbiome** | FB ratio | 14.046 | 0.001 | 0.215 | 0.948 | High (>80%) |
| **Microbiome** | Gram Negative | 9.627 | 0.008 | 0.136 | 0.761 | Moderate (60-80%) |
| **Microbiome** | Gram Positive | 9.627 | 0.008 | 0.136 | 0.761 | Moderate (60-80%) |
| **Microbiome** | Mobile elements | 10.99 | 0.004 | 0.161 | 0.842 | High (>80%) |
| **Microbiome** | Potentially Pathogenic | 9.027 | 0.011 | 0.126 | 0.719 | Moderate (60-80%) |
| **Microbiome** | Shannon Diversity | 5.725 | 0.057 | 0.067 | 0.414 | Low (40-60%) |
| **Microbiome** | Stress tolerant | 11.564 | 0.003 | 0.171 | 0.869 | High (>80%) |

**Methods**

**Sample processing for oral microbiome sequencing**

The concentration of DNA was determined by commercially available Qubit “DNA BR Kit”. For the PCR amplification, the DNA templates were diluted to concentration of 5 ng/µl in 10 mM Tris buffer (pH 8.5). The V3/V4 region of bacterial 16S ribosomal RNA (rRNA) genes was amplified using Pro341-XT (TCG-TCG-GCA-GCG-TCA-GAT-GTG-TAT-AAG-AGA-CAG-CCT-ACG-GGN-BGC-ASC-AG) and Pro805-XT (GTC-TCG-TGG-GCT-CGG-AGA-TGT-GTA-TAA-GAG-ACA-GGA-CTA-CNV-GGG-TAT-CTA-ATC-C) primers resulting in ~520 bp amplicon. Further steps for library preparation were performed based on the Illumina “16S Metagenomic Sequencing Library Preparation” protocol. Briefly, PCR clean-up, INDEX-PCR, second PCR clean-up, library quantification, normalization and pooling were performed. Bioanalyzer chip “DNA 1000” and Qubit kit were used for quantity and quality controls of each individual sample library and the final library pool. 6 pM of the final library pool were subjected to one individual sequencing run using a 600 cycle V3 kit on an Illumina MiSeq device. During the run, 946 K/mm^2^ clusters were sequenced, generating 21.4 million reads passing filter specs. Over 74.3% of the sequencing and index reads were found with a Qscore ≥ 30.

**Microbiome analysis**

16S Illumina sequencing amplicons were denoised using a function from DADA2 toolkit (version 1.18.0) ^1^. However, the full DADA2 pipeline was not implemented in this study. Operational taxonomic units (OTUs) were assigned to the denoised fragments using the sklearn classifier (version 0.23.1) ^2^ against rRNAs clustered at 99% identity from the SILVA database version 138 ^3^. Both denoising and OTU assignment were performed using the QIIME2 pipeline version 2020.11 ^4^. The OTU table was then processed using the bioconductor package phyloseq (version 1.44.0) ^5^ within R (version 4.3.1) ^6^. First, contaminant OTUs, such as those classified as chloroplast or mitochondrial, were removed from the data. The statistical significance of microbial communities at the phylum and genus level was assessed using the Wilcoxon and Kruskal-Wallis tests when comparing pairs or multiple groups, respectively, as implemented natively in R. Afterwards, differentially abundant genera were obtained using the DESeq2 R package (version 1.40.2) ^7^, via a Wald test and Benjamin-Hochberg correction for p-values (false discovery rate < 0.01). Additional significant taxa were identified using random forest classifiers, as implemented in the randomForest R package (version 4.7-1.1) ^8^, from 10,000 trees, as recommended by Roguet *et al.* (2018) ^9^. Core microbiomes were calculated and plotted following the protocol developed in the microbiome R package (version 1.22.0) ^10^. A PICRUSt analysis was then performed to predict the relative abundance of functional pathways in the microbial communities (version 2.11) ^11^, from amplicons aligned against the GreenGenes database (version 13.8) ^12^ using QIIME2. Further bacterial phenotypes were identified using BugBase ^13^. Correlations between biochemical and immunological markers were carried out via Spearman correlations against the log10- normalized OTU abundance data, according to the protocol from the microbiome R package. Associations between relevant taxa from the core microbiome, differential abundance and random forest analyses, were tested using generalized linear models, following a protocol described on the documentation of the microbiome R package. Finally, the Illumina sequencing data was deposited in the European Nucleotide Archive (ENA) database under the study accession number PRJEB82417.

**Supplementary Table S8:** **Intra-assay variability of salivary cytokine measurements across study groups.** Coefficients of variation (CVs, %) of median fluorescence intensity (MFI) are summarized as mean ± standard deviation (SD) in saliva samples from healthy (n = 20), gingivitis (n = 19) and periodontitis (n = 20) groups. Assay performance is reported as the mid-point concentration (IC_50_), coefficient of determination (R^2^) of the standard curve and the lower limit of detection (LOD). The upper limit of quantification (ULOQ) was 10,000 pg/ml.

| **Cytokines** | **Mean ± SD of CVs (%)** | | | **Assay performance** | | |
| --- | --- | --- | --- | --- | --- | --- |
|  | **Healthy (n = 20)** | **Gingivitis (n = 19)** | **Periodontitis (n = 20)** | **IC_50_** | **R^2^** | **LOD (pg/ml)** |
| **IL-4** | 9.83 ± 8.21 | 7.26 ± 9.35 | 15.32 ± 30.07 | 48.86 | 0.998 | 10.68 |
| **IL-2** | 9.52 ± 23.93 | 9.35 ± 12.50 | 11.65 ± 30.73 | 34.88 | 0.999 | 2.33 |
| **CXCL10 (IP-10)** | 15.60 ± 18.83 | 25.08 ± 21.69 | 24.22 ± 28.26 | 14.76 | 0.999 | 8.94 |
| **IL-1β** | 7.59 ± 4.95 | 13.70 ± 12.63 | 8.15 ± 6.39 | 96.24 | 0.999 | 8.85 |
| **TNF-α** | 4.27 ± 3.01 | 5.94 ± 4.26 | 10.94 ± 30.15 | 62.79 | 0.998 | 2.50 |
| **CCL2 (MCP-1)** | 11.98 ± 12.49 | 16.81 ± 21.35 | 13.71 ± 12.97 | 133.93 | 1.000 | 8.01 |
| **IL-17A** | 7.57 ± 5.39 | 10.32 ± 9.68 | 16.14 ± 30.07 | 4.19 | 0.999 | 0.66 |
| **IL-6** | 8.02 ± 6.37 | 11.01 ± 13.23 | 12.05 ± 26.58 | 25.90 | 0.999 | 3.37 |
| **IL-10** | 4.14 ± 3.11 | 6.44 ± 12.16 | 11.82 ± 30.22 | 5.27 | 0.999 | 1.05 |
| **IFN-γ** | 2.50 ± 2.06 | 3.61 ± 6.04 | 10.58 ± 27.11 | 735.82 | 0.999 | 4.57 |
| **IL-12p70** | 5.97 ± 12.48 | 7.00 ± 12.78 | 9.36 ± 29.81 | 32.98 | 0.999 | 2.46 |
| **CXCL8 (IL-8)** | 8.46 ± 5.53 | 17.20 ± 14.20 | 9.16 ± 8.32 | 279.69 | 0.997 | 1.27 |
| **TGF-β1** | 6.62 ± 5.99 | 10.33 ± 7.71 | 12.93 ± 26.47 | 1057.37 | 0.996 | 40.69 |

**Supplementary Table S9:** **Shapiro-Wilk normality tests for continuous variables.** Results of Shapiro-Wilk tests assessing normality of continuous clinical, immunological and microbiological parameters within each diagnostic group. *p* value < 0.05 indicates deviation from normal distribution. Normal distribution is indicated by “+” symbol and deviation from normality is indicated by “-“ symbol.

| **Category** | **Parameter** | **Shapiro-Wilk test (p value)** | | | **Normality** |
| --- | --- | --- | --- | --- | --- |
|  |  | **Healthy (H)** | **Gingivitis (G)** | **Periodontitis (P)** | **H/G/P** |
| **Clinical data** | Age | 0.0014 | 0.1138 | 0.5199 | - / + / + |
| **Clinical data** | API | 0.3322 | 0.5059 | 0.6466 | + / + / + |
| **Clinical data** | pCAL | <0.0001 | <0.0001 | <0.0001 | - / - / - |
| **Clinical data** | mSBI | 0.3322 | 0.5059 | 0.6466 | + / + / + |
| **Clinical data** | PD | 0.0755 | 0.0918 | 0.0010 | + / + / - |
| **Cytokines** | IL-1β | <0.0001 | 0.0648 | 0.0029 | - / + / - |
| **Cytokines** | CXCL8 (IL-8) | <0.0001 | 0.0137 | 0.0007 | - / - / - |
| **Cytokines** | CCL2 (MCP-1) | 0.2060 | 0.0018 | 0.0206 | + / - / - |
| **Cytokines** | CXCL10 (IP-10) | <0.0001 | 0.0269 | 0.0002 | - / - / - |
| **B cells** | B cells | 0.6921 | 0.5060 | 0.0684 | + / + / + |
| **B cells** | CD5^+^ B cells | 0.3563 | 0.0058 | 0.0180 | + / - / - |
| **B cells** | CD5^+^ double negative B cells | <0.0001 | 0.0248 | 0.0035 | - / - / - |
| **B cells** | CD5^+^ naive B cells | 0.3798 | 0.0220 | 0.3480 | + / - / + |
| **B cells** | CD5^+^ non-switched memory B cells | <0.0001 | 0.0041 | 0.0798 | - / - / + |
| **B cells** | CD5^+^ switched memory B cells | <0.0001 | <0.0001 | 0.0113 | - / - / - |
| **B cells** | CD5^+^ transitory B cells | 0.2838 | 0.1400 | 0.0072 | + / + / - |
| **Microbiome** | Aerobic | 0.0322 | 0.2127 | 0.0015 | - / + / - |
| **Microbiome** | Anaerobic | 0.0011 | 0.0224 | 0.0325 | - / - / - |
| **Microbiome** | Beta diversity | 0.1068 | 0.7040 | 0.2765 | + / + / + |
| **Microbiome** | Biofim-forming | 0.1282 | 0.3931 | 0.0034 | + / + / - |
| **Microbiome** | Facultatively Anaerobic | <0.0001 | 0.0239 | 0.1584 | - / - / + |
| **Microbiome** | FB ratio | 0.0018 | 0.4255 | 0.0004 | - / + / - |
| **Microbiome** | Gram Negative | 0.5078 | 0.8604 | 0.0191 | + / + / - |
| **Microbiome** | Gram Positive | 0.5078 | 0.8604 | 0.0191 | + / + / - |
| **Microbiome** | Mobile elements | 0.0025 | 0.0080 | 0.0207 | - / - / - |
| **Microbiome** | Potentially Pathogenic | 0.5121 | 0.6971 | 0.1667 | + / + / + |
| **Microbiome** | Shannon Diversity | 0.3741 | 0.4075 | 0.6688 | + / + / + |
| **Microbiome** | Stress tolerant | 0.0037 | 0.0014 | 0.0364 | - / - / - |

**References**

**1** Callahan BJ, McMurdie PJ, Rosen MJ, Han AW, Johnson AJA, Holmes SP. DADA2: High-resolution sample inference from Illumina amplicon data. *Nat Methods*. 2016;13(7):581-583. doi:10.1038/nmeth.3869.

**2** Pedregosa F, Varoquaux G, Gramfort A, et al. Scikit-learn: Machine Learning in Python. *Journal of Machine Learning Research*. 2011;(12):2825-2830.

**3** Quast C, Pruesse E, Yilmaz P, et al. The SILVA ribosomal RNA gene database project: improved data processing and web-based tools. *Nucleic Acids Res*. 2013;41(Database issue):D590-6. doi:10.1093/nar/gks1219.

**4** Bolyen E, Rideout JR, Dillon MR, et al. Reproducible, interactive, scalable and extensible microbiome data science using QIIME 2. *Nat Biotechnol*. 2019;37(8):852-857. doi:10.1038/s41587-019-0209-9.

**5** McMurdie PJ, Holmes S. phyloseq: an R package for reproducible interactive analysis and graphics of microbiome census data. *PLoS One*. 2013;8(4):e61217. doi:10.1371/journal.pone.0061217.

**6** R Core Team. R: A Language and Environment for Statistical Computing: Version 4.4.2.

**7** Love MI, Huber W, Anders S. Moderated estimation of fold change and dispersion for RNA-seq data with DESeq2. *Genome Biol*. 2014;15(12):550. doi:10.1186/s13059-014-0550-8.

**8** Liaw A, Wiener M. Classification and Regression by randomForest. *R News*. 2002;(2/3):18-22.

**9** Roguet A, Eren AM, Newton RJ, McLellan SL. Fecal source identification using random forest. *Microbiome*. 2018;6(1):185. doi:10.1186/s40168-018-0568-3.

**10** Salonen A, Salojärvi J, Lahti L, Vos WM de. The adult intestinal core microbiota is determined by analysis depth and health status. *Clin Microbiol Infect*. 2012;18 Suppl 4:16-20. doi:10.1111/j.1469-0691.2012.03855.x.

**11** Langille MGI, Zaneveld J, Caporaso JG, et al. Predictive functional profiling of microbial communities using 16S rRNA marker gene sequences. *Nat Biotechnol*. 2013;31(9):814-821. doi:10.1038/nbt.2676.

**12** DeSantis TZ, Hugenholtz P, Larsen N, et al. Greengenes, a chimera-checked 16S rRNA gene database and workbench compatible with ARB. *Appl Environ Microbiol*. 2006;72(7):5069-5072. doi:10.1128/AEM.03006-05.

**13** Ward T, Larson J, Meulemans J, et al. *BugBase predicts organism-level microbiome phenotypes*; 2017.
